# Cdk6’s functions are critically regulated by its unique C-terminus

**DOI:** 10.1016/j.isci.2024.111697

**Published:** 2024-12-27

**Authors:** Alessia Schirripa, Helge Schöppe, Sofie Nebenfuehr, Markus Zojer, Thorsten Klampfl, Valentina Kugler, Belinda S. Maw, Huriye Ceylan, Iris Z. Uras, Lisa Scheiblecker, Elisabeth Gamper, Ulrich Stelzl, Eduard Stefan, Teresa Kaserer, Veronika Sexl, Karoline Kollmann

**Affiliations:** 1 Institute of Pharmacology and Toxicology, University of Veterinary Medicine Vienna, 1210 Vienna, Austria; 2 Institute of Pharmacy/Pharmaceutical Chemistry and Center for Molecular Biosciences Innsbruck, University of Innsbruck, Innsbruck, Austria; 3 Tyrolean Cancer Research Institute (TKFI), Innrain 66, 6020 Innsbruck, Austria; 4 Institute of Molecular Biology and Center for Molecular Biosciences Innsbruck (CMBI), University of Innsbruck, Innsbruck, Austria; 5 Institute of Pharmaceutical Sciences, Pharmaceutical Chemistry, University of Graz, Graz, Austria; 6 BioTechMed-Graz, Graz, Austria; 7 Field of Excellence BioHealth - University of Graz, Graz, Austria; 8 University of Innsbruck, Innsbruck, Austria

**Keywords:** Biochemistry, Molecular Structure, Structural biology, Cancer

## Abstract

The vital cell cycle machinery is tightly regulated and alterations of its central signaling hubs are a hallmark of cancer. The activity of CDK6 is controlled by interaction with several partners including cyclins and INK4 proteins, which have been shown to mainly bind to the amino-terminal lobe. We analyzed the impact of CDK6’s C-terminus on its functions in a leukemia model, revealing a central role in promoting proliferation. C-terminally truncated *Cdk6* (Cdk6 ΔC) shows reduced nuclear translocation and therefore chromatin interaction and fails to enhance proliferation and disease progression. The combination of proteomic analysis and protein modeling highlights that Cdk6’s C-terminus is essential for protein flexibility and for its binding potential to cyclin D, p27^Kip1^ and INK4 proteins but not cyclin B. We demonstrate that the C-terminus is a unique and essential part of the CDK6 protein, regulating interaction partner binding and therefore CDK6’s functionality.

## Highlights

• Cdk6’s C-terminus is essential for protein flexibility and protein interactions
• Cdk6 ΔC shows decreased nuclear localization and chromatin interaction
• The C-terminus of CDK6 is essential for leukemia progression

## Graphical abstract

**Figure:**
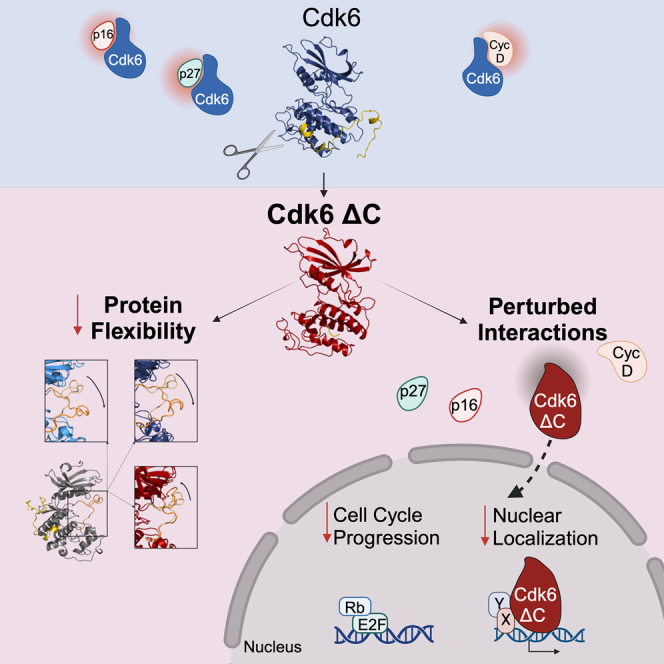

## Introduction

The family of cyclin-dependent kinases (CDKs) comprises more than 20 serine/threonine kinases functioning in diverse cellular processes including cell cycle progression, transcriptional regulation, stem cell self-renewal, DNA damage repair and metabolism.^1^ Dysregulation of CDKs is directly linked to tumorigenesis. CDK6 has received considerable attention over the past years as a major driver of cancer, including hematopoietic malignancies.^2^^,^^3^^,^^4^ Initially identified as a classical cell cycle kinase, CDK6 has also been described as a transcriptional regulator in a kinase dependent and independent manner.^5^^,^^6^^,^^7^^,^^8^ Monomeric CDKs are catalytically inactive and only when bound to cyclins they become kinase active. Upon mitogenic stimuli, CDK4 and CDK6 associate with D-type cyclins, promoting the cell cycle transition from the G1 phase to the S phase.^9^^,^^10^^,^^11^ CDK4/6-cyclin D complexes are negatively regulated by two inhibitor families. The INK4 family includes p16^INK4a^, p15^INK4b^, p18^INK4c^, and p19^INK4d^. These proteins specifically bind to monomeric CDK4 and CDK6 thus preventing the complex formation with cyclins and the activation of the kinase.^12^^,^^13^^,^^14^^,^^15^ The Cip/Kip family, including p21^Cip1^, p27^Kip1^ and p57^Kip2^, acts on a broader spectrum of CDK-cyclin complexes and inhibits CDK1, 2, 4, and 6.^16^^,^^17^^,^^18^^,^^19^ The available X-ray crystallographic structures of CDK6-inhibitor/CDK6-cyclin complexes highlight crucial sites for protein-protein interactions and complex formation.^20^^,^^21^^,^^22^^,^^23^ INK4 proteins are described to interact with the amino-terminal lobe (N-lobe) and the first part of the carboxy-terminal lobe (C-lobe) of CDK6, while the interaction with the cyclins appears to occur predominantly with the αC-helix, also known as PLSTIRE helix, at the end of CDK6’s N-lobe. While current structural models highlight the centrality of the N-lobe of CDK6 for binding of the interaction partners the role of the terminal part of the C-lobe is not well defined yet, partially due to the lack of X-ray crystallographic structures. In this study, we sought to understand the impact of the C-terminus of Cdk6 on its multiple functions by expressing a C-terminally truncated mutant (Cdk6 ΔC). The lack of the C-terminus influences Cdk6’s nuclear localization and therefore its chromatin interaction. The reduced protein flexibility of truncated Cdk6 results in an abrogation of the binding to INK4 and cyclin D proteins that does not affect the defined cyclin B interaction. The expression of truncated Cdk6 in leukemic cells leads to reduced proliferation, colony formation and longer latency compared to controls.

## Results

### The C-terminus of Cdk6 is essential for nuclear localization and chromatin interaction

**Figure 1:**
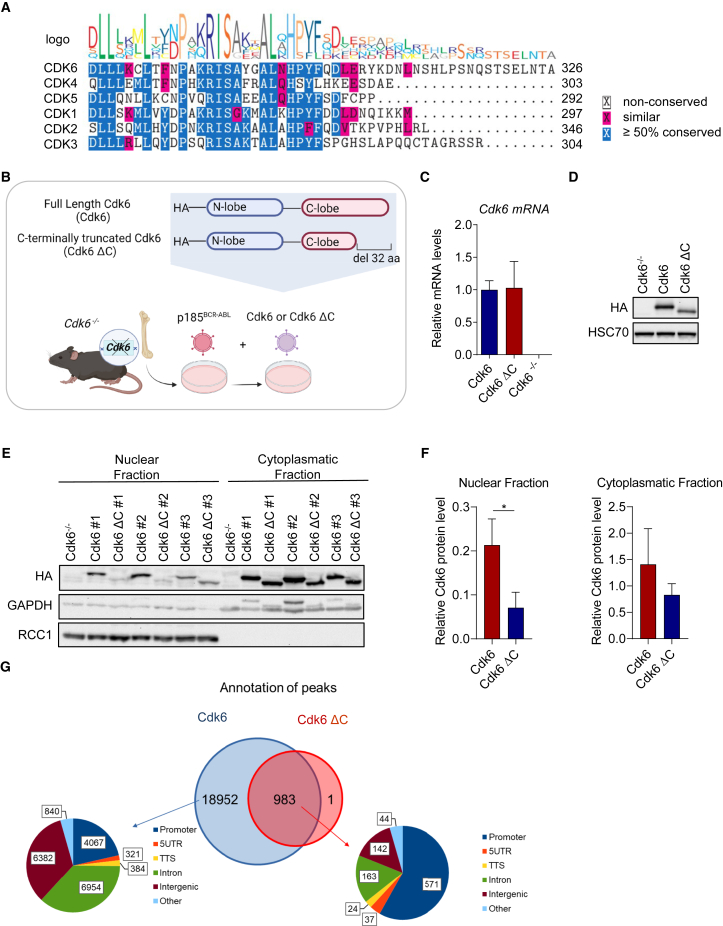
The C-terminus of Cdk6 is essential for nuclear localization and chromatin interaction (A) Alignment of the amino acid sequences of the C-terminal domains of CDKs regulating cell cycle progression. (B) Experimental scheme of the generation of stable murine *Cdk6*^−/−^ p185 BCR-ABL^+^ cell lines reconstituted with either HA-Cdk6-Full Length (Cdk6) or HA-C-terminally truncated Cdk6 (Cdk6 ΔC). (C) qPCR analysis of *Cdk6* mRNA levels of Cdk6^−/−^, Cdk6 and Cdk6 ΔC cell lines (*n* = 3 biological replicates/genotype). Levels of mRNAs were normalized to Rplp0 mRNA. Error bars show mean ± SD. (D) Western blot analysis of HA tagged Cdk6 protein levels of Cdk6^−/−^, Cdk6 and Cdk6 ΔC cell lines (representative figure of biological replicate #2). HSC70 was used as loading control. (E) Western blot analysis of nuclear/cytoplasmatic fractionation of Cdk6 and Cdk6 ΔC cell lines for HA (*n* = 3 biological replicates). GAPDH served as a cytoplasmatic marker and RCC1 as nuclear marker. (F) Western blot densitometry quantification showing relative Cdk6 protein levels in the nuclear (left) and cytoplasmatic (right) fractions compared to the respective loading control. *p* values determined by unpaired t-test (∗*p* = 0,0235). Error bars show mean ± SD. (G) Overlap of Cdk6 and Cdk6 ΔC ChIP-seq peaks detected at least in two of three samples of each genotype. The pie charts represent the functional classification of the peak regions identified in each dataset.

CDKs share a high sequence homology in the catalytic domain but have diverse functions in cell cycle and transcriptional regulation.^1^^,^^24^ Recent studies highlighted the dual role of CDK6, being a cell cycle and transcriptional regulator. An alignment of amino acid sequences of CDKs revealed the C-terminus as the region with the greatest differences between cell cycle and transcriptional CDKs (Figures 1A and S1A). The C-terminus of cell cycle CDKs is shorter compared to transcriptional CDKs. Of interest, the amino acid sequence of the C-terminus of CDK6 is evolutionarily well conserved across species with a length between cell cycle and transcriptional kinases, in line with its dual function. This prompted us to investigate the impact of the C-terminus on the multiple functions of CDK6 more closely. We took advantage of our *Cdk6*^−/−^ p185 BCR-ABL^+^ leukemic cell lines and reconstituted either a full length HA-tagged *Cdk6* (Cdk6) or a C-terminally truncated *Cdk6*, lacking the last 32 amino acids (Cdk6 ΔC) (Figure 1B). Cdk6 and Cdk6 ΔC cell lines expressed comparable *Cdk6* mRNA levels. Cdk6 ΔC showed slightly reduced protein levels compared to Cdk6 full length but comparable to endogenous Cdk6 levels (Figures 1C, 1D, S1B, and S1C). To exclude the possibility either of instability or an altered protein synthesis of the Cdk6 ΔC, we performed protein synthesis and proteasome inhibitor treatments (Figure S1D). In the absence of *de novo* protein synthesis and upon proteasome inhibition, CDK6 protein levels remained unchanged over time, irrespective of the presence or absence of the C-terminus. Albeit CDK6 is located in the cytoplasm and in the nucleus, the main function of CDK6 is attributed to its presence in the nucleus.^25^ A nuclear/cytoplasmatic fractionation showed that Cdk6 ΔC is mainly located in the cytoplasm and only a minor fraction is detectable in the nucleus compared to the full length protein (Figures 1E and 1F). We next performed chromatin immunoprecipitation sequencing (ChIP-seq) analysis in HA-Cdk6 and HA-Cdk6 ΔC expressing cell lines to understand whether the absence of the C-terminus would alter the location of chromatin binding. We identified 19,935 genomic regions to be bound by Cdk6 while only 984 regions were bound by Cdk6 ΔC. Of these 984, all with one exception (>99%) were bound by Cdk6 full length and Cdk6 ΔC. More than half of those regions were located in gene promoters (Figure 1G). Pathway enrichment analysis of the genes showed that the most highly enriched keywords were identical in both sets (Figure S1E). Locus specific comparison of Cdk6 ΔC and Cdk6 peaks revealed a lower intensity of Cdk6 ΔC peaks (Figure S1F). These findings suggest that Cdk6 ΔC maintains the ability to interact with DNA although based on the decreased nuclear availability only the sites with the strongest binding are captured. In summary, these data led us to conclude that the C-terminus of CDK6 promotes nuclear localization and thus chromatin interaction.

### Cdk6-INK4 interaction is critical for nuclear localization

**Figure 2:**
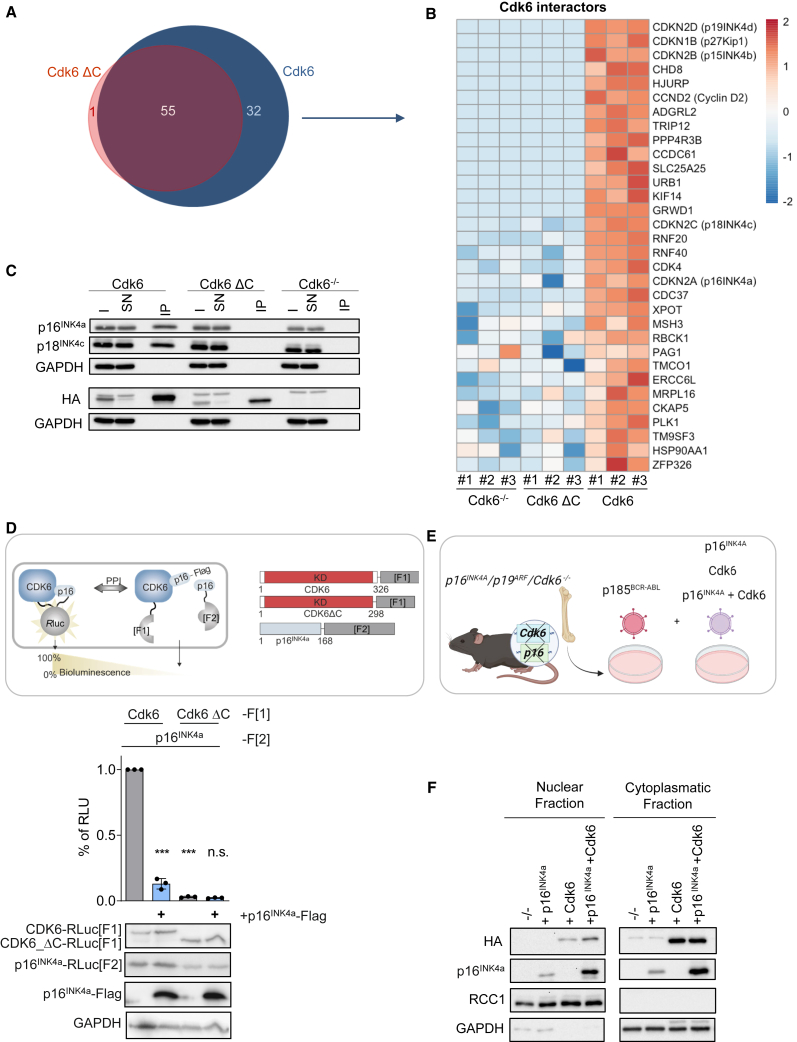
Cdk6-INK4 interaction is critical for nuclear localization (A) Venn diagram showing the numbers of interaction partners of Cdk6 and Cdk6 ΔC determined by co-immunoprecipitation (co-IP) of Cdk6 followed by mass spectrometry analysis. (B) Heatmap showing Cdk6 specific interactors. Colors indicate scaled log2 (abundance) values. For proteins with abundance levels below the detection limit (missing values), a mock value of ¼ of the smallest measured abundance in the heatmap was used. (C) Anti-HA co-IP from Cdk6, Cdk6 ΔC and Cdk6^−/−^ cell extracts analyzed for p16^INK4a^, p18^INK4c^ and HA. Cdk6^−/−^ cells served as negative control. The input (I), supernatant (SN) and immunoprecipitated (IP) fractions are shown. GAPDH served as loading control (representative figure of biological replicate #1). (D) Schematic depiction of the Rluc-PCA biosensor strategy to quantify PPIs of Cdk6 variants with p16^INK4a^ in HEK293T cells. Fragments 1 and 2 of Rluc-PCA (-F[1] and -F[2]) were fused C-terminally to p16^INK4a^ and indicated Cdk6 variants (Cdk6/Cdk6 ΔC). Protein interaction induces complementation of appended Rluc-PCA fragments. Upon addition of the Rluc substrate to intact cells bioluminescence signals are recorded (relative light units are indicated, RLU). Co-expression of flag-tagged p16^INK4a^ alters PCA interaction patterns (Error bars show mean ± SEM, *n* = 3 ind. experiments, one-sample t-test [∗*p* < 0.05, ∗∗*p* < 0.01, ∗∗∗*p* < 0.001]). (E) Experimental scheme of the generation of stable murine p16^INK4a^/p19^ARF^/Cdk6 knockout p185 BCR-ABL^+^ cell lines reconstituted with either p16^INK4a^, or Cdk6 or with both. (F) Western blot analysis of nuclear/cytoplasmatic fractionations showing HA and p16^INK4a^ protein levels in *p16*^*INK4a*^*/p19*^*ARF*^*/Cdk6*^*−/−*^ cell lines reconstituted either with p16^INK4a^, or with Cdk6, or with both. GAPDH served as a cytoplasmatic marker and RCC1 served as nuclear marker.

The reduced Cdk6 ΔC nuclear protein levels could be caused by an impaired cytoplasmatic-nuclear transport. Mechanisms to shuttle CDK6 into the nucleus are not understood yet. p21^Cip1^ and p27^Kip1^ contain a recognizable nuclear localization signal (NLS) and are involved in the shuttling of CDK4-cyclin D1 complexes to the nucleus.^26^^,^^27^^,^^28^ Therefore, we hypothesized that Cip/Kip interaction might also be relevant for CDK6’s nuclear translocation. To investigate if the C-terminus affects CDK6 interaction with its protein partners we performed a co-immunoprecipitation (coIP) of Cdk6 and Cdk6 ΔC followed by mass spectrometry analysis (IP-MS). Analysis of Cdk6 and Cdk6 ΔC binding partners revealed 55 common interactors (Figures 2A and S2A). Additionally, we found 32 protein interactors in the Cdk6 IP, that were not present in the Cdk6 ΔC pull-down (Figures 2A and 2B). p27^Kip1^ and members of the INK4 family were only found binding to full length Cdk6 (Figure 2B). The interaction with p27^Kip1^, p16^INK4a^, p18^INK4c^ and p19^INK4d^ was confirmed by immunoblotting in cells harboring full length Cdk6 but was not detectable in the Cdk6 ΔC cell lines (Figures 2C and S2B). The loss of interaction of Cdk6 ΔC with p16^INK4a^ was further validated in an independent set of experiments using a cell-based *Renilla* luciferase (Rluc) based protein-fragment complementation assay (PCA) in HEK293T cells. This reporter system has been applied successfully to study regulatory and binary interactions of kinases including PKA and BRAF.^26^^,^^29^ We thus engineered genetically encoded reporter constructs to quantify alterations of binary complex formation of p16^INK4a^: Cdk6 in intact cells (see scheme in Figure 2D). No Cdk6 ΔC-p16^INK4a^ interaction was observed but a strong signal for Cdk6-p16^INK4a^ was detected confirming the reduced ability of Cdk6 ΔC to bind p16 ^INK4a^ (Figure 2D). To determine if the Cdk6-p16^INK4a^ interaction accounts for the nuclear localization of Cdk6 we made use of p185 BCR-ABL^+^ *Cdkn2a*^*−/−*^/*Cdk6*^*−/−*^ cells (*p16* ^*INK4a*^*/p19*^*ARF*^*/Cdk6*^*−/−*^) cells. These cells were reconstituted with p16^INK4a^ or HA-Cdk6 or both (Figure 2E)*.* The nuclear/cytoplasmatic fractionation of the cells showed increased nuclear Cdk6 levels in cells that concomitantly express p16^INK4a^ (Figures 2F and S2C). This finding underscores the role of p16^INK4a^ for the nuclear localization of Cdk6. The absence of p16^INK4a^ did not completely abrogate the presence of Cdk6 in the nucleus that may be explained by the involvement of additional proteins. We propose that the p27^Kip1^ interaction with CDK6 is at least partially responsible for CDK6 nuclear translocation, as it was shown for CDK4,^27^ and that the missing interaction with Cdk6 ΔC reduces its nuclear shuttling. These results indicate that the CDK6 C-terminus is indispensable for complex formation with p27^Kip1^ and INK4 proteins that drives nuclear shuttling.

### Cdk6’s C-terminus is essential for D-type cyclin binding

**Figure 3:**
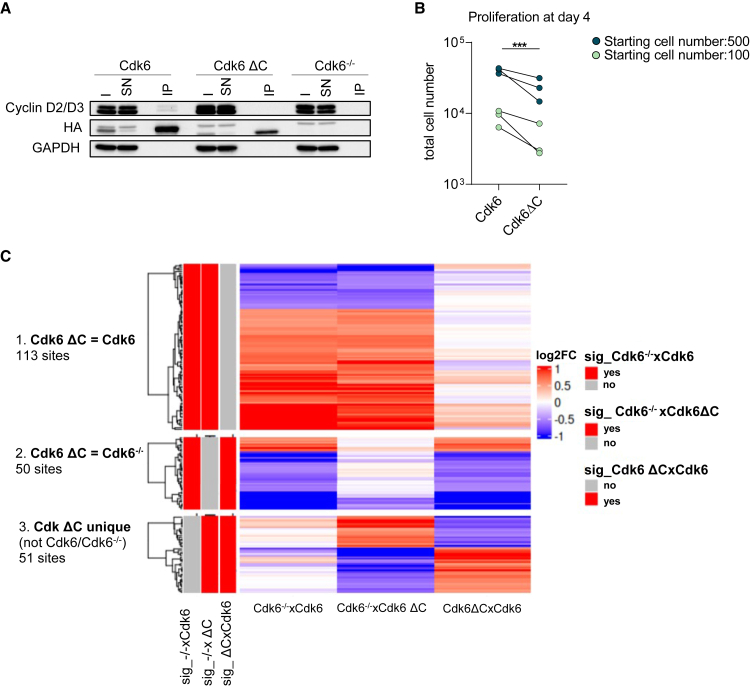
Cdk6 C-terminus is essential for D-type cyclin binding (A) Anti-HA co-IP from Cdk6, Cdk6 ΔC and Cdk6^−/−^ cell extracts analyzed for cyclin D2/D3 and HA. Cdk6^−/−^ cells served as negative control. The input (I), supernatant (SN), and immunoprecipitated (IP) fractions are shown. GAPDH served as loading control (biological replicate #1). (B) Cell numbers at day 4 of the growth curve (*n* = 3 biological replicates, *p* = 0.000973). (C) Phospho-chromatome analysis of Cdk6, Cdk6 ΔC and Cdk6^−/−^ cell lines: results were grouped based on peptide phosphorylation status relative to Cdk6 ΔC (Cdk6 ΔC = Cdk6, Cdk6 ΔC = Cdk6^−/−^, Cdk6 ΔC unique; *n* = 3 biological replicates). Colors indicate fold changes in phosphorylation of individual peptides.

Cyclin D and INK4 binding was predicted to occur via the N-lobe of CDK6. As we observed an impaired ability of Cdk6 ΔC to form complexes with the INK4 proteins, we closely analyzed the interaction with cyclin D proteins in the IP-MS analysis (Figure 2B). coIP experiments confirmed that cyclin D2 and D3 interaction was abrogated when the C-terminus of Cdk6 is truncated (Figures 3A and S3A). In line, cell proliferation was impaired in Cdk6 ΔC cells compared to Cdk6 cells (Figures 3B and S3B). Cyclin D binding is essential for CDK6 kinase activity that prompted us to analyze the phosphorylation sites of chromatin-bound proteins by a phospho-chromatome analysis. Three main clusters were identified (Figure 3C) as follows. (1) One cluster of phospho-sites changed similarly in Cdk6 and Cdk6 ΔC cells when compared to *Cdk6*^*−/−*^ cells. This could be explained by a compensatory mechanism of other kinases in the Cdk6 ΔC cells. Interestingly, we found cyclin B bound to Cdk6 ΔC and Cdk6 in the IP-MS data that could also at least partially explain this commonly regulated phospho-sites (Figure S2A). Cyclin B typically interacts with CDK1 in the M-phase of the cell cycle.^28^ (2) A second cluster consisted of phospho-sites similar in Cdk6 ΔC and Cdk6^−/−^ cells but changed in Cdk6 cells. These phospho-sites might be specific targets of CDK6-cyclin D complexes. (3) A third group of phospho-sites uniquely changed in the Cdk6 ΔC cells. For a better characterization, we overlapped those sites with data from a publicly available systematic substrate screen for CDK4 and CDK6 from Anders et al.^30^ Among the substrates that show reduced phosphorylation uniquely in the Cdk6 ΔC compared to Cdk6 and Cdk6^−/−^ cells was FOXM1, a known leukemic regulator (Figure S3C). These results highlight an at least partially dominant negative function of Cdk6 ΔC for tumor promotion. All in all, C-terminally truncated Cdk6 alters the phosphorylation pattern in cells underlining a reduced kinase activity.

### The C-terminus of Cdk6 is essential for leukemia progression

**Figure 4:**
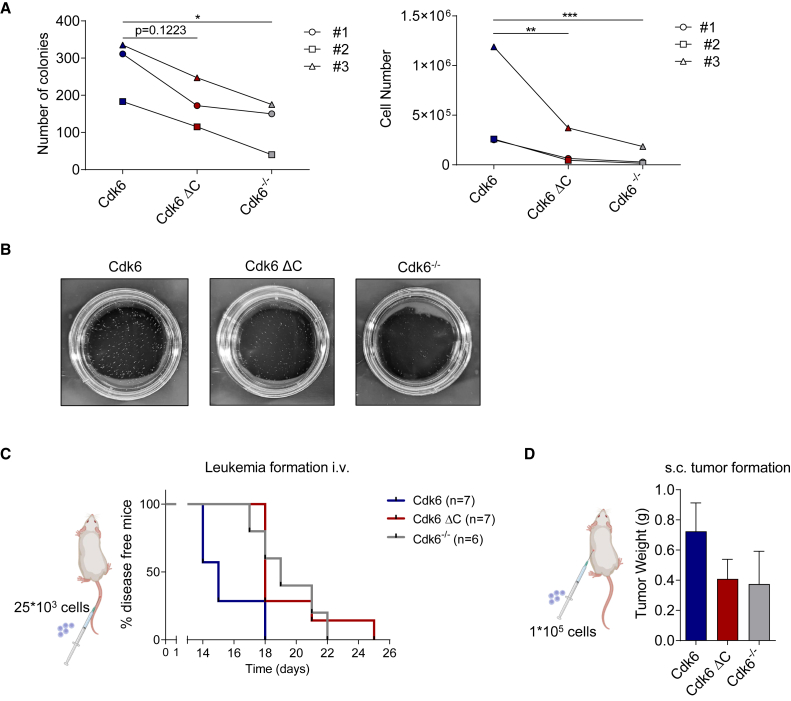
The C-terminus of Cdk6 is essential for leukemia progression (A) Colony-formation assays (CFAs) of cells expressing either Cdk6, Cdk6 ΔC or no Cdk6 in growth factor–free methylcellulose. Number of colonies per dish (left) and total cell count (right) after incubation for 7 days is shown (*n* = 3 biological replicates). Figure 4A, left: Cdk6 vs. Cdk6 ΔC *p*-value = 0.1223. Cdk6 vs. Cdk6^−/−^*p*-value = 0.0282. Figure 4A, right: Cdk6 ΔC vs. Cdk6 *p*-value = 0.0019. Cdk6^−/−^ vs. Cdk6 *p*-value = 0.0006. (B) Representative macroscopic pictures of CFA of Cdk6, Cdk6 ΔC or Cdk6^−/−^ cell lines. (C) Kaplan-Meier-plot of NSG mice intravenously injected with 25 × 10^3^ cells expressing either Cdk6, Cdk6 ΔC or no Cdk6. Cdk6 *n* = 7, Cdk6 ΔC *n* = 7, Cdk6^−/−^*n* = 6. Cdk6 vs. Cdk6 ΔC ∗∗*p* = 0.0057, Cdk6 vs. Cdk6^−/−^ ∗*p* = 0.0161. (D) 1 × 10^5^ Cdk6, Cdk6 ΔC or Cdk6^−/−^ cells were injected subcutaneously into NSG mice. Tumor weight was determined after 8 days of injection (Cdk6 *n* = 6, Cdk6 ΔC *n* = 6, Cdk6^−/−^*n* = 4). Error bars show mean ± SEM.

To assess the biological relevance of CDK6’s C-terminus for clonogenic cell growth, we performed a colony formation assay. A defined number of cells were embedded into growth factor-free methylcellulose and after 7 days colonies were counted (Figures 4A, 4B, and S4A). In accordance with our published data showing that CDK6 overexpression does not lead to a massive proliferative advantage due to p16^INK4a^ induction and in turn CDK6 inhibition,^31^ we observed minor alterations in the colony formation assay. The expression of Cdk6 ΔC, although not binding to INK4 proteins, failed to increase colony numbers and size. This finding confirms the limited kinase function of the Cdk6 ΔC. To further evaluate the impact of the truncated Cdk6 on leukemia progression, we injected our different cell lines intravenously into NSG mice. Consistently with the *in vitro* observation the disease latency of mice injected with cells carrying the truncated Cdk6 was similar to mice injected with Cdk6 knockout cells (Figure 4C). Enlarged spleens at the day of euthanasia in all mice validated the presence of disease (Figure S4B). In line, Cdk6 ΔC cells showed similar tumor formation potential as Cdk6 knockout cells when implanted subcutaneously into mice (Figure 4D). CDK6 regulates blood vessel formation in subcutaneous tumors.^31^ Tumors derived from cells expressing truncated Cdk6 showed no increase in vascularization compared to tumors lacking Cdk6 (Figure S4C). In summary, C-terminally truncated Cdk6 in leukemic cells is not able to enhance proliferation and vascularization.

### The C-terminus of Cdk6 determines protein flexibility

**Figure 5:**
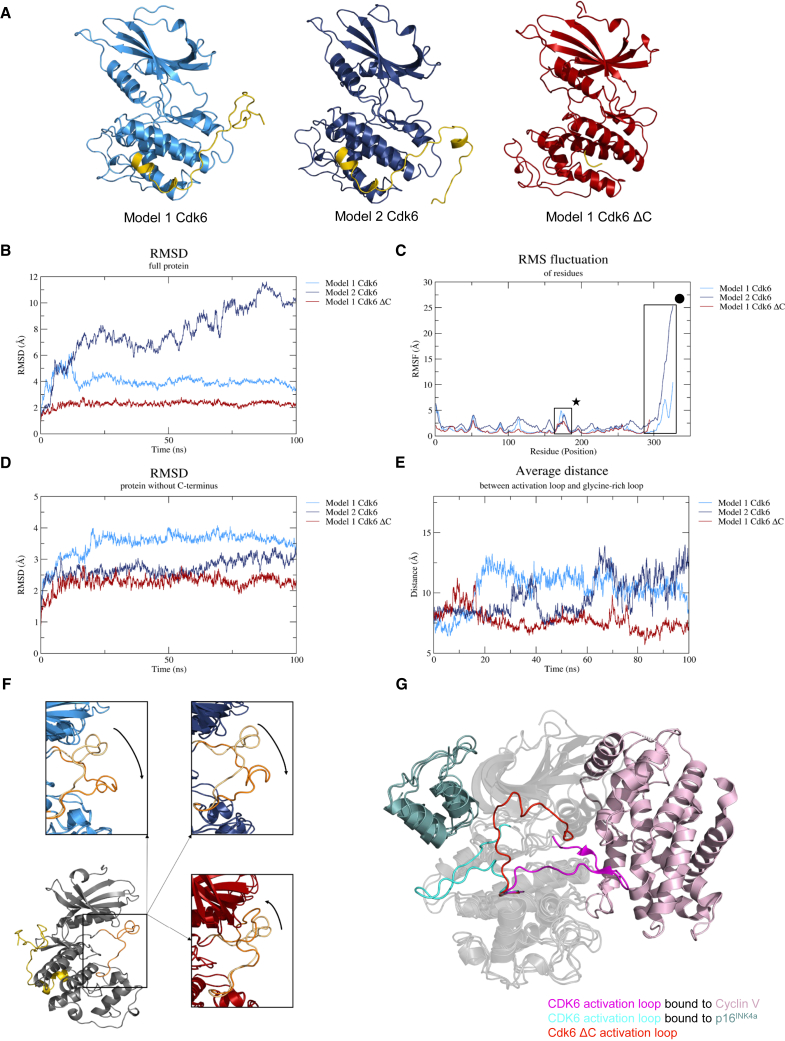
The C-terminus of Cdk6 determines protein flexibility **(**A) Structures of the two Cdk6 (light and dark blue) and the Cdk6 ΔC (red) homology models with C-termini colored yellow. (B) The root-mean-square deviation (RMSD) of the Cdk6 models (light and dark blue) show a much higher deviation of the input structure in the time course of the molecular dynamics (MD) simulation compared to model 1 ΔC (red). (C) The root-mean-square fluctuation (RMSF) analysis of the MD simulation results of the Cdk6 models indicate a much higher mobility of the activation loop (highlighted with ★) residues compared to model 1 ΔC. The C-termini residues (highlighted with •) of both Cdk6 models show the highest level of motion in the time course of the MD simulation. (D) The RMSD of the protein without the C-terminus is decreased for model 2 Cdk6 in comparison of the full protein (B) highlighting the contribution of the C-terminus to the flexibility of the protein. Due to the lack of the C-terminus in model 1 Cdk6 ΔC no differences were observed. (E) The average distance between the activation loop and the glycine-rich loop of Cdk6 models vary to a much higher extent compared to model 1 Cdk6 ΔC during the MD simulation. (F) The input structure of model 1 Cdk6 (gray) with activation loop and C-terminus colored in orange and yellow, respectively. The analysis of the MD simulation trajectories revealed an outward movement of the Cdk6 model activation loop (orange; indicated by the arrows, insets in light and dark blue) in comparison to the starting structure (light orange). For model 1 Cdk6 ΔC (inset in red), the activation loop moves in the opposite direction indicating a more inactive conformation. (G) Alignment of the homology model 1 Cdk6 ΔC (activation loop in red) with Cdk6 (activation loop in cyan) in complex with p16^INK4a^ (light teal, PDB entry 1BI7^20^) and Cdk6 (activation loop in magenta) bound to V-cyclin (light pink, PDB entry 1XO2^40^) shows the different activation loop conformations that are required to interact with the different binding partners.

We next set out to investigate Cdk6 and Cdk6 ΔC protein structures with the aim to gain a better understanding of the impact of the C-terminus at the molecular level. Of note, all currently available experimental CDK6 structures lack the complete C-terminus and analyses using PSIPRED,^32^^,^^33^ DisoFlag,^34^ MetaDisorder,^35^^,^^36^ and AIUPred^37^ predicted that C-terminal residues were disordered. Therefore, homology models of Cdk6 and Cdk6 ΔC structures were generated, based on the structure of CDK6 in complex with p19^INK4D^ (PDB: entry 1BLX ^21^). Structural comparison of related CDKs revealed that in many structures the longer C-terminus adopts loop conformations, whereas for CDK9 also a helical conformation was observed (Figure S5A). We selected the C-terminus of CDK2 (PDB entry 3PXF ^38^) as a template for an extended CDK6 C-terminus in our models. The two highest-scoring structures of the resulting Cdk6 models contained both a C-terminus in a loop (model 1 Cdk6, Figure 5A left panel) and a helical (model 2 Cdk6, Figure 5A, middle panel) conformation that have been selected for further analysis. For Cdk6 ΔC, only the highest-ranked model (model 1 Cdk6 ΔC, Figure 5A, right panel) was chosen. Of note, experimental structures of CDK6 in complex with cyclins or INKs show no indication that the C-terminus directly interacts with those proteins (Figure S5B). This suggests that the observed loss of binding was not due to the loss of direct protein-protein interactions with the C-terminus of Cdk6. To investigate the impact of protein dynamics, all models were subjected to 100 ns long molecular dynamics (MD) simulations. This revealed a much higher deviation from the starting structure as indicated by the higher root-mean-square deviation (RMSD), and thus much larger conformational changes for both Cdk6 models in comparison to the Cdk6 ΔC model (Figure 5B). An analysis of the root-mean-square fluctuation (RMSF), a parameter investigating the flexibility of individual residues, highlighted that residues in the C-terminus of the Cdk6 models were highly mobile (Figure 5C). To investigate the contribution of the C-terminus on overall protein flexibility, the RMSD was re-calculated for the kinase domain core only (i.e., without the C-terminus) (Figure 5D). In the case of model 1 Cdk6, the RMSD remains at a similar level of around 4 Å compared to the full protein (Figure 5B). However, the RMSD of the kinase core in model 2 Cdk6 is considerably decreased (Figure 5D), suggesting that the C-terminus accounts for the majority of the flexibility observed for the full protein. Intriguingly, we observed another set of residues, where the flexibility was higher in the Cdk6 models compared to the Cdk6 ΔC model (Figure 5C). These residues form the activation loop of the kinase, which is a major determinant of protein function. In the input conformations for the MD simulation, the activation loop is folded across the front of the ATP binding site, and it is located in close proximity to the glycine-rich loop. The activation loop thereby blocks access to the binding site and this conformation is thus indicative of a catalytically inactive kinase conformation. Given that the activation loop residues appear to be more flexible in the Cdk6 models (Figure S5C), we investigated whether this affected the distance between the activation loop and the glycine-rich loop and consequently access to the binding site (Figure 5E). For the Cdk6 ΔC model, the distance remained largely unchanged around 7.5 Å, with the exception of peaks around 10 and 70 ns. In contrast, the distance between activation and glycine-rich loop increased considerably in model 1 Cdk6, where closer proximity is only observed toward the end of the simulation. Similarly, the two loops move apart in model 2 Cdk6, with intermittent conformations between 40 and 60 ns where they are located in closer proximity. Toward the end of the simulation, the distance between activation- and glycine-rich loop continuously increases. Consistent, structural analyses of the MD simulation trajectories of both Cdk6 models revealed the presence of more open activation loop conformations (Figure 5F; Videos S1 and S2), which facilitate access to the ATP binding site and are thus associated with active kinase conformations (Figure 5G). In contrast, the Cdk6 ΔC activation loop blocks access to the ATP binding site throughout the simulation (Video S3). In some frames, it moves even closer toward the glycine-rich loop (Figure 5F). Similar results were retrieved when the simulation of model 1 Cdk6 ΔC was repeated (Figures S5D and S5E). Taipale et al. demonstrated that the interaction of kinases with the HSP90 and CDC37 (co-)chaperones is disrupted when kinase conformations are stabilized by inhibitor binding^39^ suggesting that chaperone binding is a general indicator of kinase flexibility. Intriguingly, our IP-MS analysis revealed loss of HSP90 and CDC37 interactions for Cdk6 ΔC (Figure 2B) that is in line with the reduced flexibility observed in the MD simulations. Alignment of experimental Cdk6 structures bound to p16^INK4a^ (i.e., inactive conformation, PDB entry 1BI7^20^) and cyclin V (i.e., active conformation, PDB entry 1XO2^40^) to the Cdk6 ΔC model demonstrates that different activation loop conformations are required to allow binding of different interaction partners (Figure 5G). We therefore hypothesize that the plasticity observed for the activation loop in the Cdk6 models is induced by the presence of the flexible C-terminus and that this is a critical prerequisite required for many protein-protein interactions and thus Cdk6 functions. In contrast, the limited activation loop flexibility observed for Cdk6 ΔC prevents the kinase to adopt conformational changes required for binding to many of its binding partners.

Video S1. Structural analyses of the MD simulation trajectories of Cdk6 model 1, showing the presence of more open activation loop conformations, related to Figure 5F

Video S2. Structural analyses of the MD simulation trajectories of Cdk6 model 2, showing the presence of more open activation loop conformations, related to Figure 5F

Video S3. Structural analyses of the MD simulation trajectories of Cdk6 ΔC revealing that the activation loop blocks access to the ATP binding site throughout the simulation. In some frames, it moves even closer toward the glycine-rich loop, related to Figure 5F

### The C-terminus of Cdk6 is not essential for cyclin B1 interaction

**Figure 6:**
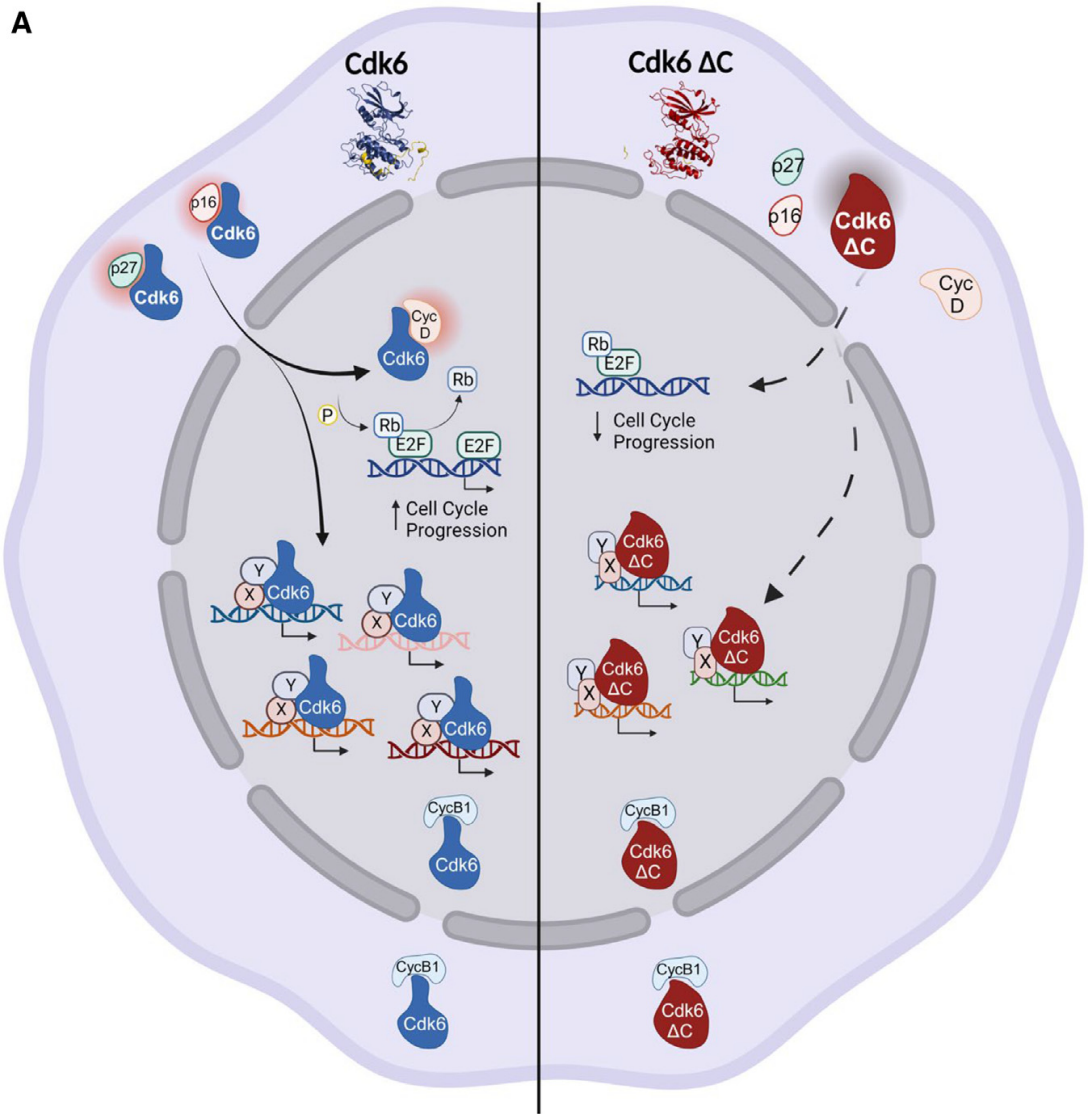
Model (A) Graphical representation of our model displaying the consequences of a shorter C-terminal domain on Cdk6 functions. The shorter C-terminal domain of Cdk6 ΔC reduces its protein flexibility. This in turn causes the loss of Cdk6 ΔC interactions with partners crucial for cell cycle progression (cyclin D) and for its nuclear translocation (p27^Kip1^ and p16^INK4a^), therefore perturbing its chromatin interactions. The binding of cyclin B is not affected by the C-terminus of Cdk6.

One notable exception presents cyclin B1, which was detected to interact with both Cdk6 and Cdk6 ΔC (Figure S2A). Experimental heterodimeric structures of CDK6 in complex with a cyclin are only available for the viral cyclin V (e.g., PDB entries 1XO2,^40^ 1JOW,^23^ and 2EUF^41^). Interestingly, comparison with the CDK2-cyclin B1 complex (PDB entry 2JGZ^42^) revealed a similar protein fold and binding mode of cyclin B1 and cyclin V (Figure S6A). In the absence of an experimental CDK6-cyclin D structure, the closely related CDK4-cyclin D1 complex (PDB code 2W9Z^43^) was aligned to CDK6-cyclin V and showed that the CDK6-cyclin V interface differs and is much larger (FigureS6B). Buried interaction surface areas of 2,334 Å^2^ for CDK4-cyclin D1^43^ and 3,974 Å^2^ for CDK6- cyclin V^43^ have been reported. The smallest surface area was observed for CDK6-p19^INK4d^,^21^ which only comprised 1,700 Å^2^ (Figure S6B). We hypothesized, that due to the larger interaction interface, cyclin B1 can stabilize the Cdk6 conformation required for cyclin B1 binding even in the absence of the C-terminus. In contrast, the smaller interaction interface observed for cyclin D1 potentially leads to weaker interactions, which are more easily disrupted and not sufficient to stabilize the Cdk6 conformation required for cyclin D1 binding in Cdk6 ΔC.

### The C-terminus of Cdk6 affects access to the ATP binding site

Finally, we investigated whether the Cdk6 ΔC model is still compatible with ligand binding, using palbociclib (PDB code 5L2I^44^) as an example. We hypothesized that the reduced folding of the activation loop might affect availability of the ligand binding site. On top, also additional residues within the ligand binding site adopted conformations that clashed with palbociclib binding (Figure S7A). This concept was tested by incubation of Cdk6 and Cdk6 ΔC harboring cells with BSJ-03-123, a palbociclib-based PROTAC (Figure S7B).^45^ Protein levels of full length Cdk6 were decreased upon treatment with BSJ as expected (Figure S7B), indicating successful target engagement. In contrast, BSJ-mediated degradation was absent in cells harboring Cdk6 ΔC consistent with the loss of palbociclib binding. All in all, we propose a model where the shorter C-terminal domain of Cdk6 ΔC reduces its protein flexibility (Figure 6A). This in turn causes the loss of its interactions with crucial regulating partners and ligands and abrogates its functions.

## Discussion

The CDK family members play critical roles in several cellular processes such as cell cycle control and transcription. Therefore, it is not surprising that deregulation of CDK activity is a hallmark of cancer.

The high degree of sequence homology suggests that CDK structures are relatively similar. All CDKs consist of a two-lobed structure possessing an N-lobe rich in β-sheets and a C-lobe containing α-helices as well as an active site located at the interface between the two lobes.^24^ The sizes of the CDKs range from approximately 250 amino acids, which just comprise the catalytic domain, to more than 1,000 residues. Sequence alignments of different CDKs displayed that the C-terminus is the site that differs the most in length and amino acid composition. It appears that CDKs, having functions as cell cycle regulators, are composed of a shorter C-terminal amino acid sequence compared to those that modulate gene transcription where the C-terminus is much longer. Interestingly, CDK6 is the cell cycle CDK with the longest C-terminus and this might already point at its function in modulating transcription, placing CDK6 in the transition between the cell cycle associated CDKs and the transcriptional ones. Available structures of CDK6 in complex with its characterized activating and inhibiting regulators display regions like the CDK6 N-lobe and only a limited fragment of the C-lobe to be primarily involved in the binding with the interactors.^20^^,^^21^^,^^22^^,^^23^^,^^41^ Our results shed new light on the vital importance of the C-lobe for CDK6’s functions and structure. We demonstrate that a shorter C-terminus perturbs CDK6’s protein flexibility and abrogates its interaction with known partners that are fundamental for its functional regulation. Our data show a reduced nuclear localization of the C-terminally truncated Cdk6 that is at least partially due to the abrogation of the binding to the INK4 and Cip/Kip family members. This is in line with the characterized mechanism for CDK4 that shuttles into the nucleus upon p21^Cip1^/p27^Kip1^ binding.^27^^,^^46^ A recent study suggested that CDK6 gets transported to the nucleus via a piggy-back mechanism on cargo proteins containing classical NLSs.^6^ We propose a model where CDK6 nuclear shuttling is not only guaranteed by its binding to p27^Kip1^ but also by the binding to p16^INK4a^. Despite INK4 proteins do not contain obvious NLSs, it has been reported that some proteins can use independent nuclear transport mechanisms.^47^ Missense mutations of specific p16^INK4a^ residues that are directly involved in the interaction with the N-lobe of CDK6, such as D74 and D84, have been frequently found in cancers. Substitutions at these residues significantly decrease CDK4/6-inhibitory activities.^48^^,^^49^^,^^50^ Similarly, the point mutation CDK4^R24C^ in hereditary melanoma patients abolishes the interaction with p16^INK4a^ leading to elevated CDK4 kinase activity.^48^ So far, the homologous R31C variant of CDK6 has not been found in patients, maybe due to the more complex CDK6-p16^INK4a^ interaction. Our data might propose a scenario where less CDK6 is shuttled to the nucleus in patients harboring p16^INK4a^ mutations or deletions. This could imply that CDK6’s functions in the cytoplasm are enhanced while nuclear functions are decreased that might lead to a different signaling pattern and the need for precise therapeutic strategies. However, further investigations are needed to shed light on the changes of CDK6’s function in the absence and presence of p16^INK4a^. Besides the perturbed INK4 interaction, the deletion of the C-terminus of Cdk6 also impairs its interaction with cyclin D proteins and therefore suggests a limited kinase activity. Structural modeling implies that the C-terminus confers higher protein flexibility, which is necessary to adopt the diverse conformations required for binding to the different protein partners and to allow access to the ATP binding site. Interestingly, we find three main patterns when analyzing phospho-chromatome data. One set of phospho-sites showed a similar pattern for Cdk6 ΔC and Cdk6 compared to Cdk6^−/−^ cells. This result might be explained by a regulation via other kinases that is independent of the C-terminus of CDK6. Another explanation would be given by the fact that cyclin B binds to Cdk6 and Cdk6 ΔC that could lead to CDK6 kinase activity. This result together with the computational modeling suggests that cyclin B binding to CDK6 does not resemble cyclin D binding to CDK6. Please note, however, that in the absence of experimental CDK6-cyclin B1 and CDK6-cyclin D1 structures, we cannot exclude that CDK6-bound cyclin B1 and cyclin D1 adopt different binding modes compared to CDK2 and CDK4. In addition, more experiments are needed to understand which effects come from a direct CDK6-cyclin B interaction or an indirect compensation in Cdk6 ΔC cells. Overall, Cdk6 ΔC resulted in reduced phospho-sites when compared to Cdk6 and even to Cdk6^−/−^ cells, pointing at a suppressive function of Cdk6 ΔC. Cdk6 ΔC might block phospho-sites that are consequently not accessible to other kinases. Of note, we found the phosphorylation of FOXM1, a known CDK4/6 substrate,^30^ to be particularly diminished in Cdk6 ΔC cell lines. In the past years, FOXM1 has been shown to be involved in different aspects of tumorigenesis including suppression of cellular senescence.^51^ In particular, FOXM1 upregulation has been reported in BCR-ABL1^+^ acute lymphoblastic leukemia (ALL) where its deletion impairs cell proliferation and viability as well as leukemia formation.^52^ This might be one underlying mechanism why Cdk6 ΔC shows a tumor-suppressive phenotype compared to Cdk6. Given the fact that CDK4/6 kinase inhibitors are already used in clinics but show a number of limitations when treating certain types of cancers and the appearance of resistance mechanisms to CDK4/6 kinase inhibitors, new strategies to target CDK6 are needed.^53^^,^^54^ Our findings draw the attention to the C-terminal lobe of CDK6 as an innovative exploitable target for the development of drugs that interfere with CDK6’s kinase dependent and independent functions. However, intensive research is still needed to understand the consequences of inhibiting the C-terminus of CDK6 for a whole organism.

### Limitations of the study

One caveat of the study might be the overexpression system in the Cdk6^−/−^ p185 BCR-ABL^+^ stable cell lines, which potentially masks some effects of endogenously expressed Cdk6 and Cdk6 ΔC. The cell lines might already have adapted some compensatory mechanisms, e.g., p53 mutations, which could interfere with the effects of the mutant Cdk6. To evaluate comprehensive effects of Cdk6 ΔC, analysis of cell lines expressing endogenous Cdk6 and Cdk6 ΔC should be conducted. Further, our study only addresses the effects of Cdk6 ΔC in leukemogenesis; however, it would also be important to evaluate the impact of Cdk6 ΔC on normal hematopoiesis. It is likely that the perturbed Cdk6 ΔC functionality and the loss of interactions with its binding partners influences hematopoietic stem cell activity.

Understanding whether and how the binding affinity of Cdk6 for ATP is altered by the truncation of its C-terminus remains unresolved and would require the purification of recombinant Cdk6 and Cdk6 ΔC proteins to perform microscale thermophoresis (MST) assays. Recombinant proteins would also help to clarify the kinase activity of the Cdk6 ΔC in complex with cyclin D and cyclin B. Of note, previous studies suggest kinase activity for Cdk6 bound to INK4 proteins, which underlines a kinase activity also independent of cyclin D. However, an *in vitro* kinase assay may not fully reflect the physiological kinase activity of Cdk6, as this setting lacks cellular influences such as localization, feedback mechanisms and protein-protein interactions. Additionally, the choice of substrate in this assay can impact result accuracy, and it needs to be determined if complex-dependent substrates exist.

## Resource availability

### Lead contact

Further information and requests for resources and reagents should be directed to and will be fulfilled by the lead contact, Karoline Kollmann (karoline.kollmann@vetmeduni.ac.at).

### Materials availability

All the materials generated in this study will be available upon request from the lead contact.

### Data and code availability

• ChIP-seq data have been deposited at ArrayExpress and are publicly available as of the date of publication. Accession numbers are listed in the key resources table. Mass spectrometry and Phosphoproteome data have been deposited at Proteomics Identifications Databases (PRIDE) and are publicly available as of the date of publication. Accession numbers are listed in the key resources table.
• This paper does not report original code.
• Any additional information required to reanalyze the data reported in this paper is available from the lead contact upon request.

## Acknowledgments

We thank S. Fajmann, P. Jodl, and Petra Kudweis for their excellent technical support. We would also like to thank Karin Hummel and the Proteomics VetCore Facility of the University of Veterinary Medicine Vienna (Vienna, Austria) for performing and supporting the IP-MS experiment. The computational results presented here have been achieved (in part) using the LEO HPC infrastructure of the University of Innsbruck. Graphics were created with BioRender.com.

This work was supported by the 10.13039/501100000781European Research Council (ERC) under the 10.13039/501100007601European Union’s Horizon 2020 research and innovation program grant agreement no 694354 (V.S.). This research was funded in whole or in part by the 10.13039/501100002428Austrian Science Fund (FWF) SFB-F6107 (V.S.), the 10.13039/501100002428Austrian Science Fund (FWF) P31773 (K.K.), the 10.13039/501100002428Austrian Science Fund (FWF) P34376 (T. Kaserer), the 10.13039/501100002428Austrian Science Fund (FWF) P30441 (E.S.), the 10.13039/501100002428Austrian Science Fund (FWF) P32960 (E.S.), the 10.13039/501100002428Austrian Science Fund (FWF) P35159 (E.S.), and the 10.13039/501100002428Austrian Science Fund (FWF) I5406 (E.S.). For open access purposes, the author has applied a CC BY public copyright license to any author-accepted manuscript version arising from this submission.

## Author contributions

Conceptualization and design: A.S., S.N., V.S., and K.K. Acquisition of data: A.S., H.S., S.N., T. Kaserer, V.K., B.S.M., H.C., I.Z.U., L.S., and E.G. Analysis and interpretation of data: A.S., H.S., S.N., T. Kaserer, V.K., M.Z., T. Klampfl, U.S., E.S., and K.K. Writing of the manuscript: A.S., H.S., T. Kaserer, and K.K. Study supervision: V.S. and K.K. All authors have read and agreed to the published version of the manuscript.

## Declaration of interests

The authors declare no competing interests.

## STAR★Methods

### Key resources table

**Table:** 

REAGENT or RESOURCE	SOURCE	IDENTIFIER
**Antibodies**		

Rabbit polyclonal anti-CDK6	Santa Cruz Biotechnology	Cat# sc-7180; RRID: AB_2076998
Mouse monoclonal anti-HSC70	Santa Cruz Biotechnology	Cat# sc-7298; RRID: AB_627761
Rabbit polyclonal anti-CDK4	Santa Cruz Biotechnology	Cat# sc-260; RRID: AB_631219)
Rabbit recombinant monoclonal anti-GAPDH	Cell Signaling Technology	Cat# 8884; RRID: AB_11129865
Mouse monoclonal anti-RCC1	Santa Cruz Biotechnology	Cat# sc-55559; RRID: AB_831160
Mouse monoclonal anti-β-actin	Santa Cruz Biotechnology	Cat# sc-69879; RRID: AB_1119529
Rabbit polyclonal anti-HA tag	Abcam	Cat# ab9110; RRID: AB_307019
Rabbit polyclonal anti-p27^Kip1^	Proteintech	Cat# 26714-1-AP; RRID: AB_2880611
Rabbit recombinant monoclonal anti-p16^INK4a^	Abcam	Cat# ab211542; RRID: AB_2891084
Rabbit recombinant monoclonal anti-p18^INK4c^	Abcam	Cat# ab192239; RRID: AB_3674728
Rabbit polyclonal anti-p19^INK4d^	Thermo Fisher Scientific	Cat# PA5-26413; RRID: AB_2543913
Rabbit monoclonal anti-cyclin D2	Santa Cruz Biotechnology	Cat# sc-593; RRID: AB_2070794
Rabbit polyclonal anti-cyclin D3	Santa Cruz Biotechnology	Cat# sc-182; RRID: AB_2259653
Mouse monoclonal anti-Flag® M2	Sigma-Aldrich	Cat# F3165; RRID: AB_259529
Rabbit recombinant monoclonal anti-GAPDH	Cell Signaling Technology	Cat# 2118; RRID: AB_561053
Mouse monoclonal anti-Renilla Luciferase F1	Sigma-Aldrich	Cat# MAB4410-I; RRID: AB_3674729
Rabbit monoclonal anti-Renilla Luciferase F2	Abcam	Cat# ab185926; RRID: AB_3083537

**Chemicals, peptides, and recombinant proteins**		

Pierce™ Anti-HA Magnetic Beads	ThermoFisher Scientific	Cat# 88837
DSG (disuccinimidyl glutarate)	ThermoFisher Scientific	Cat# 20593
Pierce™ 16% Formaldehyde (w/v), Methanol-free	ThermoFisher Scientific	Cat# 28906
Dynabeads™ Protein G for Immunoprecipitation	Invitrogen	Cat# 10003D
Cycloheximide	Sigma-Aldrich	Cat# C1988
Epoxomycin	Gentaur Molecular Products	Cat# 607-A2606
Pierce™ BCA Protein Assay Kits	ThermoFisher Scientific	Cat#23225
Trypsin/Lys-C Mix, Mass Spec Grade	Promega	Cat# V5071
TransFectin™ Lipid Reagent	Bio-Rad	Cat# 1703352
Coelenterazine h	NanoLight Technology	Cat# 301
BSJ-03-123	MedChemExpress LLC	Cat# HY-111556
Mouse Methylcellulose Base Media	R&D Systems	Cat# HSC006
Cytiva SpeedBeads magnetic carboxylate modified particles A (hydrophobic)	Sigma-Aldrich	GE65152105050250
Cytiva SpeedBeads magnetic carboxylate modified particles B (hydrophilic)	Sigma-Aldrich	GE45152105050250

**Critical commercial assays**		

RNeasy MiniKit	QIAGEN	Cat# 74104
iScript™ cDNA synthesis kit	Bio-Rad	Cat# 1708890
SsoAdvanced Universal SYBR Green Supermix	Bio-Rad	Cat# 1725270
Clarity™ Western ECL Substrate	Bio-Rad	Cat# 1705060
20X LumiGLO® Reagent and 20X Peroxide	Cell Signaling	Cat# 7003
Pierce™ Bradford Protein Assay Kit	ThermoFisher Scientific	Cat# 23200
NEBNext® Ultra™ II DNA Library Prep Kit for Illumina®	New England Biolabs	Cat# E7645S

**Deposited data**		

ChIP-Seq data	This paper	ArrayExpress: E-MTAB-13798 (https://www.ebi.ac.uk/biostudies/arrayexpress/studies/E-MTAB-13798)
Mass spectrometry data	This paper	Proteomics Identifications Databases (PRIDE): PXD058474 (https://www.ebi.ac.uk/pride/archive/projects/PXD058474)
Phosphochromatome data	This paper	Proteomics Identifications Databases (PRIDE): PXD059037 (https://www.ebi.ac.uk/pride/archive/projects/PXD059037)

**Experimental models: Cell lines**		

Cdk6^-/-^ BCR/ABL^+^ cell lines	This paper	N/A
HA-Cdk6 BCR/ABL^+^ cell lines	This paper	N/A
HA-Cdk6 -ΔC BCR/ABL^+^ cell lines	This paper	N/A
Cdkn2a^-/-^/Cdk6^-/-^ BCR/ABL^+^ cell lines	This paper	N/A
Cdkn2a^-/-^/HA-Cdk6 BCR/ABL^+^ cell lines	This paper	N/A
p16^INK4a^/Cdk6^-/-^ BCR/ABL^+^ cell lines	This paper	N/A
HEK293T	ATCC	CRL-3216

**Experimental models: Organisms/strains**		

Cdk6^-/-^ mice	Malumbres M. et al.^55^	N/A
p16^INK4a^/p19^ARF^/Cdk6^-/-^ mice	MGI ID: 1857942	Cdkn2a<tm1Rdp> Targeted Allele Detail MGI Mouse (MGI:1857942) (jax.org)
NSG mice	NOD.Cg-Prkdc^scid^ Il2rgtm^1Wjl^/SzJ	The Jackson Laboratory

**Oligonucleotides**		

Cdk6 fwd, 5'-GCTTCGTGGCTCTGAAGCGCG-3'	This paper	N/A
Cdk6 rev, 5'-TGGTTTCTGTGGGTACGCCGG-3'	This paper	N/A
p16^INK4a^ fwd, 5'-GTGTGCATGACGTGCGGG-3'	This paper	N/A
p16^INK4a^ rev, 5'-GCAGTTCGAATCTGCACCGTAG -3'	This paper	N/A
Rplp0 fwd, 5’-AGATTCGGGATATGCTGTTGG-3’	This paper	N/A
Rplp0 rev, 5’-AAAGCCTGGAAGAAGGAGGTC-3’	This paper	N/A

**Software and algorithms**		

Proteome Discoverer software version 2.4.1.15	ThermoFisher Scientific	XCALI-98057
R programming language	R Foundation	N/A
Molecular Operating Environment (MOE) version 2020.0901	Chemical Computing Group	https://www.chemcomp.com/en/index.htm
Maestro Schroedinger release 2022-3	Maestro, Schrödinger, LLC, New York, NY, 2023	https://www.schrodinger.com
Gromacs version 2022.4	Gromacs development team	https://manual.gromacs.org
CHARMM36 force field version July 2022	Huang et al.^56^	https://mackerell.umaryland.edu/charmm_ff.shtml#gromacs
Grace-5.1.25	Grace Development Team Maintained by Evgeny Stambulchik	https://plasma-gate.weizmann.ac.il/pub/grace/
VMD version 1.9.4a55	Humphrey et al.^57^	https://www.ks.uiuc.edu/Development/Download/download.cgi?PackageName=VMD
PyMol version 2.5.0	The PyMOL Molecular Graphics System, Version 2.5.0 Schrödinger, LLC	https://pymol.org
Proteome Discoverer software version 2.4.1.15	ThermoFisher Scientific	N/A
FASTQC	Babraham Bioinformatics	https://www.bioinformatics.babraham.ac.uk/projects/fastqc
Trimmomatic (v0.36)	Bolger et al.^58^	http://www.usadellab.org/cms/index.php?page=trimmomatic
bwa-mem (v0.7.15)	Li et al.^59^	https://github.com/lh3/bwa
bedtools subtract (v2.26.0)	Quinlan et al.^60^	https://github.com/arq5x/bedtools
samtools (v1.3.1)	Danecek et al.^61^	https://github.com/samtools/samtools/releases/download/1.3.1/samtools-1.3.1.tar.bz2
MACS2 (v2.1.0)	Zhang et al.^62^	https://pypi.org/project/MACS2/
Homer (v4.9.0)	Heinz et al.^63^	http://homer.ucsd.edu/homer/
GraphPad Prism 5.04	GraphPad Software	N/A

### Experimental model and study participant details

#### Mice

*Cdk6*^*-/-*^^55^ and NSG mice (NOD.Cg-Prkdc^scid^ Il2rgtm^1Wjl^/SzJ; The Jackson Laboratory) were bred and maintained under specific pathogen-free conditions at the Institute of Pharmacology and Toxicology, University of Veterinary Medicine Vienna. All animal experiments were conducted in 8- to 10-week-old mice. Balanced numbers of males and females were used in all experiments, enabling us to test for any effects that might be sex-specific. All procedures were performed in accordance with protocols approved by the Austrian law and the Animal Welfare Committee at the University of Veterinary Medicine Vienna (Licence: BMBWF-68.205/0174-V/3b/2018).

#### Cell lines

Preparation of retroviral supernatant and generation of *Cdk6*^*-/-*^ p185 BCR-ABL^+^ and Cdkn2a^-/-^/Cdk6^-/-^ p185 BCR/ABL^+^ cell lines and the re-expression of either HA-Cdk6 or HA-Cdk6-ΔC in *Cdk6*^*-/-*^ p185 BCR-ABL^+^ cell lines and of either HA-Cdk6 or p16^INK4a^ in Cdkn2a^-/-^/Cdk6^-/-^ p185 BCR/ABL^+^ cell lines was performed as previously described.^31^^,^^64^ Cell lines were cultured in RPMI medium supplemented with 10% heat inactivated fetal bovine serum (FBS), 50 μM 2-mercaptoethanol and 100 U/ml penicillin, 100 μg/ml streptomycin.

### Method details

#### RNA extraction and qPCR analysis

Total RNA was isolated from stable cell lines using the RNeasy MiniKit (Qiagen, Venlo, Netherlands) as recommended by the manufacturer’s instructions. Reverse transcription was performed using the iScript cDNA synthesis kit (Bio-Rad, Hercules, CA, USA). All qPCRs were performed in duplicate with the SsoAdvanced Universal SYBR Green Supermix (Bio-Rad, Hercules, CA, USA) according to the instructions of the manufacturer on a CFX96 Real-Time System C1000Touch Thermal Cycler (Bio-Rad). Primer sequences for *Cdk6* were as follows: fwd, 5'-GCTTCGTGGCTCTGAAGCGCG-3' and rev, 5'-TGGTTTCTGTGGGTACGCCGG-3'. Primer sequences for *p16* ^*INK4a*^ were as follows: fwd, 5'-GTGTGCATGACGTGCGGG-3' and rev, 5'-GCAGTTCGAATCTGCACCGTAG -3'. Levels of mRNAs were normalized to *Rplp0* mRNA.

#### Western blot analysis and immunoprecipitation (IP)

Cell lysis and blotting were performed as previously described.^65^ Membranes were blocked in 5% BSA followed by incubation with primary antibodies: anti-HA (ab9110, Abcam), anti-CDK6 (H-96, Santa Cruz), anti-HSC70 (B-6, Santa Cruz), anti-CDK4 (C-22, Santa Cruz), anti-GAPDH (D16H11, Cell Signaling Technology), anti-RCC1 (E-6, Santa Cruz), anti β-actin (AC-1, Santa Cruz). Secondary HRP antibodies (Cell Signaling Technology, Danvers, MA, USA) were used and membranes were visualized using Clarity™ ECL Western blotting substrate (Biorad, Hercules, CA, USA) or 20X LumiGLO® Reagent and 20X Peroxide (Cell Signaling Technology). Immunoprecipitation of HA-tagged Cdk6, using Pierce™ Anti-HA Magnetic Beads (Thermo Fisher Scientific) were used and the following antibodies were used for detection: anti-GAPDH (D16H11, Cell Signaling Technology), anti-HA (ab9110, Abcam), anti-p27^KIP1^ (26714-1-AP, ThermoFisher Scientific), anti-p16 ^INK4a^ (ab211542, Abcam), anti-p18 ^INK4c^ (ab192239, Abcam), anti-p19 ^INK4d^ (PA5-26413, ThermoFisher), anti-cyclinD2/D3 (M-20/C-16, Santa Cruz).

#### Nuclear and cytoplasmic cell fractionation

20x10^6^ cells were washed with PBS and centrifuged for 4 min, 400 x g at 4°C. Cell pellets were resuspended in 200 μl buffer A (10 mM N-2-hydroxyethylpiperazine-N'-2-ethanesulfonic acid (HEPES) pH 7.9 (Sigma-Aldrich), 0.1 mM EDTA (Carl Roth), 0.1 mM EGTA (Sigma), 2 mM DTT (Sigma), 25 mM NaF (Merck), 1 mM PMSF (Sigma), 0.4 mM Na_3_VO_4_ (Sigma), 10 mM KCl (Carl Roth), 20 μg/ml leupeptin (Sigma), 20 U/ml aprotinin (Sigma)). Samples were incubated for 15 min on ice and 10% NP-40 (0.6% final concentration) was added to the cells. Samples were vortexed for 10 seconds and then centrifuged for 60 sec, 13.000 g, 4°C. The cytoplasmic supernatant was collected into a new 1.5 ml tube and stored at -80°C. The nuclear pellet was washed six times with 1 ml ice cold PBS and centrifuged for 1 min with 13.000 x g at 4°C. 100 μl buffer B (20 mM HEPES pH 7.9, 25% glycerol, 0.4 mM Na-vanadate, 400 mM NaCl, 1 mM EGTA, 2 mM DTT, 1 mM EDTA, 25 mM Na-fluoride, 1 mM PMSF, 20 μg/ml leupeptin, 20 U/ml aprotinin) was added to the nuclear pellets. Samples were vigorously mixed on the shaker for 45 min at 4°C and centrifuged (20 min, 13.000, 4°C) afterwards. The supernatant (soluble nuclear fraction) was collected and stored at -80°C. Protein concentrations were measured using Bradford Assay Kit (Thermo Fischer Scientific).

#### ChIP-seq

Cdk6 or Cdk6 ΔC chromatin immunoprecipitation (ChIP) was performed using an antibody against HA (ab9110, Abcam) as described previously.^64^^,^^66^ Three cell lines per genotype were used. Cells were crosslinked with DSG (20 minutes, RT) and 1% formaldehyde (10 minutes, RT) and termination of the fixation procedure was performed using glycine. For immunoprecipitation (IP), 70 μl Dynabeads Protein G magnetic beads (Invitrogen) were used. IP DNA as well as corresponding input DNA were subjected to sequencing library preparation (NEBNext Ultra II chemistry, New England Biolabs) and sequencing was performed on an Illumina HiSeq2500 sequencer. Raw sequencing reads were quality controlled using FASTQC followed by quality filtering, trimming of reads and adapter removal using trimmomatic (v0.36).^58^ Mapping against the mouse reference genome (Gencode M13) was done using bwa-mem (v0.7.15)^59^ and blacklisted regions were removed using bedtools subtract (v2.26.0).^60^ Multimappers and reads with bad mapping quality were removed using samtools (v1.3.1).^61^ Peak calling was performed by MACS2 (v2.1.0)^62^ using default parameters. In order to call a genomic region to be bound by Cdk6 or Cdk6 DC we required peaks to be found in that region in at least 2 out of 3 replicate cell lines. Motif identification was performed using Homer (v4.9.0)^63^ findMotifsGenome.pl with the default -size 200. ChIP-seq data are deposited in the Gene Expression Omnibus (GEO) database.

#### Pathway enrichment analysis

Enrichment for UniProt keywords was tested in lists of genes that either had only Cdk6 ChIP Seq peaks in their promotors or that had both, Cdk6 and Cdk6 ΔC ChIP Seq peaks. The analysis was performed using STRING enrichment analysis (string-db.org).

#### CDK6 protein stability and degradation

To determine Cdk6 protein stability and degradation, Cdk6 and Cdk6 ΔC cells were treated either with 40 μg/ml of cycloheximide (Chx, Sigma-Aldrich, C1988) to inhibit translational elongation or with 10 μM of the proteasome inhibitor Epoxomicyn (Epox, Gentaur Molecular Products, 607-A2606). At indicated time points, whole cell lysates were prepared and analyzed by western blot.

#### Immunoprecipitation coupled to mass spectrometry analysis

Cell lysis was performed using RIPA lysis buffer composed of 150 mM NaCl, 50 mM Tris (pH=8), 0,1% SDS, 0,5% NaDOC, 1% NP-40 in the presence a protease inhibitor cocktail (Roche, Basel, Switzerland). Protein concentration was measured colorimetrically (Pierce™ BCA Protein Assay Kit, Thermo Fisher Scientific, Waltham, MA, USA) on an EnSpire® Multimode Plate Reader (Perkin Elmer, Waltham, MA, USA). 1 mg of protein and 35 μl of the Pierce™ Anti-HA Magnetic Beads (Thermo Fisher Scientific) were incubated overnight at 4°C under rotation. Protein– Anti-HA Magnetic Beads complexes were washed 5 times with RIPA buffer. For the elution of the protein complexes, samples were solved in 25 μl IP elution buffer (50 mM HEPES, 150 mM NaCl, 5 mM EDTA, 2% SDS, 1x proteinase inhibitor). For reduction and alkylation 1.4 μl of 200 mM tris-(2 carboxyethyl)-phosphine (TCEP) in 100 mM triethylammonium bicarbonate (TEAB) as well as 1.4 μl of 800 mM chloroacetamide (CAA) in 100 mM TEAB were added. Reduction and alkylation were performed on the thermomixer for 25 min at 60°C. After cool-down, proteins were loaded onto 4 μl magnetic bead solution corresponding to 200 μg beads (Cytiva SpeedBeads magnetic carboxylate modified particles A (hydrophobic) and B (hydrophilic) in a 1:1 ratio). Then 75 μl ethanol absolute were added for protein binding to the beads, followed by eight washing steps each with 160 μl 80% ethanol. After transfer of protein/bead solution to a fresh tube, two additional washing steps with 180 μl ethanol were applied in order to remove remaining SDS. After a final wash with 180 μl ACN, beads were air-dried. For tryptic digest beads were resuspended in 70 μl 100 mM TEAB and 2 μl of Trypsin/LysC Mix (Promega) were added. Digest proceeded for 14 hours at 37°C. Digested peptides were collected using the magnetic rack and beads re-extracted twice with 50 μl 100 mM TEAB. Before LC-MS analysis combined, acidified peptide extracts were desalted using C18 spin tips (Pierce, Thermo Fisher) according to the manufacturers protocol. Peptides dissolved in 12 μl 0.1% TFA and 3 μl sample were analyzed in two technical replicates using a nanoRSLC-nESI-QExactive-Orbitrap HF MS/MS system (Thermo Fisher Scientific) as described in Gutiérrez et al.^67^ Raw data evaluation was performed with Proteome Discoverer software (version 2.4.1.15, Thermo Fisher Scientific). For database search a combination of the UniProt mouse database (taxonomy 10090, download March 2023, www.uniprot.org) and a common contaminant database (https://www.thegpm.org/crap/, accessed on 25 June 2019) was used. Search parameters were applied as follows: enzyme trypsin (full); maximally 2 missed cleavages; 10 ppm precursor mass tolerance and 0.02 Da fragment mass tolerance; dynamic modifications allowed were oxidation/+15.995 Da (M)/deamidation/+0.984 Da (N, Q)/Gln->pyro-Glu/−17.027 Da (Q) and static modification carbamidomethylation/+57.021 Da (C). For intensity-based label-free quantification, protein abundance raw values were extracted in Proteome Discoverer software (Thermo Fisher) followed by normalization to total area sums and aggregation of protein abundances of technical replicates by the mean. After filtering and exclusion of proteins with one or two missing values per group, we analyzed the remaining proteins with the intent to identify specific binding partners of Cdk6 and/or Cdk6 ΔC. We did that in two batches: First, proteins with missing abundance values: Those proteins for which we had abundance measurements only in Cdk6 samples but not in Cdk6 ΔC and Cdk6^-/-^ samples were regarded as specific binding partners of Cdk6 only. Likewise, proteins with abundance measures in Cdk6 and Cdk6 ΔC samples but not in Cdk6^-/-^ were regarded as specific binding partners of both Cdk6 variants. Second, for the remaining proteins (for which we had abundance values in all samples) we performed differential binding analysis by modelling normalized abundance as a function of genotype using a linear model in R. Multiple testing correction was performed by adjusting the P values from the linear model with the false discovery rate method. In addition to the afore mentioned proteins, we regarded proteins with an adjusted P value of < 0.2 and an absolute fold-change of < -4 in the comparison Cdk6 ΔC vs Cdk6 as further candidates for Cdk6 binding partners. Likewise, we regarded proteins with adjusted P value of < 0.2 and an absolute fold-change of > 4 in the comparisons Cdk6 ΔC vs Cdk6^-/-^ and Cdk6 vs Cdk6^-/-^ as further candidates for Cdk6 and Cdk6 ΔC binding partners.

#### Luciferase PCA analyses

Renilla luciferase (Rluc) based protein-fragment complementation assays (PCA) were performed similarly as previously described.^26^^,^^29^ Genetically encoded constructs were generated by cloning of the desired protein sequences (accession numbers: mouse CDK6: Q64261; mouse p16^INK4a^: P51480) into pcDNA3.1 expression vectors to the 5` region of RLuc fragments -F[1] or -F[2] and an interjacent linker sequence (as previously described). Thus, we engineered genetically encoded reporter constructs for quantifications of alterations of binary complex formation of p16 ^INK4a^:Cdk6 in intact cells. The schematic illustration for the used Rluc-PCA PPI reporter system is shown in Figure 2D. We fused the previously described Rluc-PCA fragments to the C-terminus of p16 ^INK4a^ and the two Cdk6 variants, delivering Cdk6-F[1], Cdk6 ΔC-F[1] and p16 ^INK4a^ -F[2]. For the competition experiments we generated an expression construct delivering the hybrid protein p16 ^INK4a^ -flag. HEK293T cells were grown in DMEM supplemented with 10% FBS. The indicated RLuc- tagged constructs were transiently overexpressed following transfection of HEK293T cells with TransFectin reagent (Bio-Rad, #1703352) grown in a 24-well plate format. Forty-eight hours after transfection the medium was carefully aspirated, and the cells were washed with PBS. Cell suspensions were transferred to white walled 96-well plates and subjected to bioluminescence analysis using the PHERAstar FSX (BMG labtech, Ortenberg, Germany). Luciferase signals (relative light units, RLU) were integrated for 10 seconds following addition of luciferase substrate benzyl-coelenterazine (Nanolight, #301). Immunoblotting was performed to determine the expression levels of the overexpressed constructs. Antibodies used: Monoclonal ANTI-FLAG® M2 antibody (Sigma Aldrich; F3165-1MG); GAPDH (Cell Signaling; 2118S); Anti-Renilla Luciferase F1 (Millipore; MAB4410); Recombinant Anti-Renilla Luciferase F2 (Abcam; ab185926).

#### Phosphochromatome

Chromatome analyses were performed as described previously.^64^ Briefly, the purified chromatin pellet was subjected to Benzonase digestion and solubilized in SDS lysis buffer. Filter Aided Sample Prep (FASP) was performed according to the procedure described previously (Wisniewski et al., 2009). Peptides were desalted using C18 solid phase extraction spin columns (The Nest Group, Southborough, MA) labelled with TMT 6plex™ reagents (Pierce, Rockford, IL) and pooled. Organic solvent was removed in vacuum concentrator and labelled peptides were loaded onto a solid phase extraction column. Peptides were eluted with 300 μL 80% acetonitrile containing 0.1% trifluoroacetic to achieve a final peptide concentration of ∼1μg/μl. Eluate was then used for phosphopeptide enrichment applying a modified method of immobilized metal affinity chromatography (IMAC) (Ficarro et al., 2005). Briefly, two times 100 μL of Ni-NTA superflow slurry (Qiagen) were washed with LCMS-grade water and Ni2+ stripped off the beads by incubation with 100 mM of EDTA, pH 8 solution for 1 h at room temperature. Stripped NTA resin was recharged with Fe3+-ions by incubation with a fresh solution of Fe(III)Cl3 and 75 μL of charged resin used for the enrichment of a total of ∼300 μg TMT-labelled peptide. The unbound fraction was transferred to a fresh glass vial and used for offline fractionation for the analysis of the whole chromatome proteome. After washing the slurry with 0.1% TFA, phosphopeptides were eluted with a freshly prepared ammonia solution containing 3mM EDTA, pH 8 and all used for offline fractionation for the analysis of the phosphoproteome. Offline fractionation via RP-HPLC at pH 10 and 2D-RP/RP Liquid Chromatography Mass Spectrometry were performed as described.^64^ Raw data files were processed using the Proteome Discoverer 2.2.0. platform, utilizing the Sequest HT database search engine and Percolator validation software node (V3.04) to remove false positives with a false discovery rate (FDR) of 1% on peptide and protein level under strict conditions. Searches were performed with full tryptic digestion against the mouse SwissProt database v2017.12 (25 293 sequences and appended known contaminants) with up to two miscleavage sites.^64^ As for the comparability of different mass spectrometry runs, we included a merged sample on all runs, to which all samples are normalized. For statistical analysis and p-value calculation, we exported the normalized abundance values from PD, corrected these values by normalizing to the merged sample and imported the resulting values into R where the log2 fold changes and p-values (using the t-test function of R) between the groups Cdk6, Cdk6 ΔC and Cdk6^-/-^ were calculated. We did not perform a multiple testing correction as the independence of the measurements cannot be guaranteed.

#### Cell proliferation assay

Either 100 or 500 cells were sorted in a well of a 96 well plate U-bottom for each time point to determine the proliferation parameters. The counts were performed every 48 hours for 8 days using a Cytoflex S (Beckman Coulter) flow cytometer. Data were analyzed using CytExpert for data analysis. In order to assess differential growth between Cdk6 and Cdk6 ΔC we chose to analyze cell counts from day 4 as this day represented the logarithmic growth phase of the cultures. We modelled the log10 of the cell counts from day 4 as a function of genotype and cell line. Genotype and cell line were encoded as two- and three-level factors, respectively, with Cdk6 and cell line#1 as reference levels. The log10 of the different starting numbers of the two experiments (100 and 500 cells) were included in the model as an offset variable. The model was fitted using the lm() function from the stats package in R version 4.0.3.

#### Structure generation and preparation

For the generation of the input structures, homology models were created using the default settings of Molecular Operating Environment version 2020.0901,^68^ except that C-terminal and N-terminal Outgap Modeling was permitted. The structure of CDK6 in complex with p19^INK4D^ (PDB entry 1BLX ^21^) was used as template for the majority of the kinase domain and CDK2 (PDB entry 3PXF ^38^) served as template for a longer C-terminus. The identical protein sequences of Cdk6 Full length and Cdk6 ΔC used in the experimental part were used to construct the models. The top two and highest ranked output structures of Cdk6 and Cdk6 ΔC were selected, respectively. All structures were prepared using the default settings of the protein preparation wizard in Maestro release 2022-3 (Maestro, Schrödinger, LLC, New York, NY, 2023).

#### Molecular dynamics simulation and data analysis

For the molecular dynamic simulation GROMACS^69^^,^^70^ version 2022.4 was used. The protein, solvent and ions were parameterized using CHARMM36 force field^56^^,^^71^ version July 2022. The simulations were performed in a cubic box with a minimum distance to the box boundaries of 10 Å, the systems were solvated with SPC/E^72^^,^^73^ water molecules and neutralized with sodium ions. After the system was minimized, it was equilibrated with 100 ps runs using both NVT and NPT ensemble where N = 69385 (Model 1 CDK6), 82939 (Model 2 CDK6) and 66013 (Model 1 CDK6 ΔC); T= 300K and P= 1 bar were. Each production run was simulated over 100 ns using time steps of 2 fs. Output frames were saved every 10 ps. The temperature was maintained at 300 K and the pressure was maintained at 1 bar, both values were controlled by modified Berendsen thermostat^74^ and the Parrinello-Rahman barostat.^75^ Bond parameters were defined by the LINCS^76^ algorithm and long-range electrostatics by the Particle-Mesh Ewald^77^ algorithms. The analysis of the results was performed using the GROMACS analysis tools (rms, rmsf, average distance) and the histograms were generated in Grace-5.1.25 (Copyright (©) 1991-1995 Paul J Turner, Portland, OR; Copyright (©) 1996-2007 Grace Development Team Maintained by Evgeny Stambulchik). The videos were produced in VMD version 1.9.4a55^57^ and figures were created using Pymol version 2.5.0 (The PyMOL Molecular Graphics System, Version 2.5.0 Schrödinger, LLC).

#### CDK6 degrader treatments

BSJ-03-123 (BSJ) was purchased from MedChemExpress LLC (Princeton, NJ, USA).^45^ BSJ was used at concentration of 3 μM. Cells were seeded for the treatments at a density of 0.4 × 10^6^ cells per mL and analyzed after 6 hours of treatment.

#### Colony formation assay

A defined number of cells was embedded into growth factor–free methylcellulose (Mouse Methylcellulose Base Media, HSC006, R&D Systems). After 7 days, colonies were counted using an inverted microscope device (CKX41, Olympus) and collected for total cell count. In order to assess differential colony formation between Cdk6, Cdk6 ΔC and Cdk6^-/-^ we modelled the log10 of the colony counts (or the total cell number) as a function of genotype and cell line. Both independent variables were encoded as three-level factors with Cdk6 WT and cell line #1 as reference levels, respectively. The models were fitted using the lm() function from the stats package in R version 4.0.3. Multiple hypothesis testing correction was performed using the p.adjust() function with method = “fdr”.

#### Tumor cell implants

To analyze leukemogenesis, 25 × 10^3^ cells from independently derived p185 BCR-ABL^+^ Cdk6, Cdk6 ΔC and Cdk6^-/-^ cell lines were intravenously injected into NSG mice. Mice were monitored daily for symptoms of disease (ruffled coat, hunched back, weakness, reduced motility) to determine the time of killing for animals with signs of distress. Sick mice were euthanized and the presence of leukemic cells in BM, spleen and blood was assessed by FACS analysis. P values determined by Mantel-Cox test and Gehan-Breslow-Wilcoxon test. To study subcutaneous tumor formation, 1 × 10^5^ cells were injected into the flank of NSG mice. Mice were euthanized on day 8 and tumor weights were analyzed. Immunofluorescence staining for blood vessels was performed and analyzed as previouslydescribed in.^31^ P values determined by paired one-way ANOVA test (∗∗∗∗p<0,0001). Error bars show mean ± s.d.

### Quantification and statistical analysis

All the experiments were performed in three biological replicates with the following exceptions: nuclear/cytoplasmatic fractionation of *p16*^*INK4a*^*/p19*^*ARF*^*/Cdk6*^*-/-*^ cell lines reconstituted with p16^INK4a^, or with Cdk6, or with both (n=1 biological replicate), cycloheximide and epoxomicin treatments (n=1 biological replicate), BSJ-03-123 treatment (n=1 biological replicate). All data are presented as the mean values with the SD, except for the Rluc-PCA biosensor to quantify PPIs where data are presented as mean ± SEM. All statistical analyses were performed using GraphPad Prism 5.04 (GraphPad) software. The number of independent biological replicates (n) is indicated in the figure legends. For macroscopic/microscopy pictures of the colony formation assay and spleen pictures, the reported images are representative of three independent biological replicates. A detailed description of quantification and statistical analysis performed to analyze ChIP-Seq, mass-Spectrometry, phospho-chromatome, cell proliferation and the colony formation assay is included in each respective method paragraph.
