# Supplementary material for: Cdk6’s functions are critically regulated by its unique C-terminus
Source: iScience. 2024 Dec 27;28(2):111697. doi: 10.1016/j.isci.2024.111697 (PMC11787673; doi:10.1016/j.isci.2024.111697)
Supplement: Document S1. Figures S1–S7 [file mmc1.pdf]

iScience, Volume *28*

Supplemental information

Cdk6's functions are critically regulated

by its unique C-terminus

Alessia Schirripa, Helge Schöppe, Sofie Nebenfuehr, Markus Zojer, Thorsten Klampfl, Valentina Kugler, Belinda S. Maw, Huriye Ceylan, Iris Z. Uras, Lisa Scheiblecker, Elisabeth Gamper, Ulrich Stelzl, Eduard Stefan, Teresa Kaserer, Veronika Sexl, and Karoline Kollmann

Fig. S1

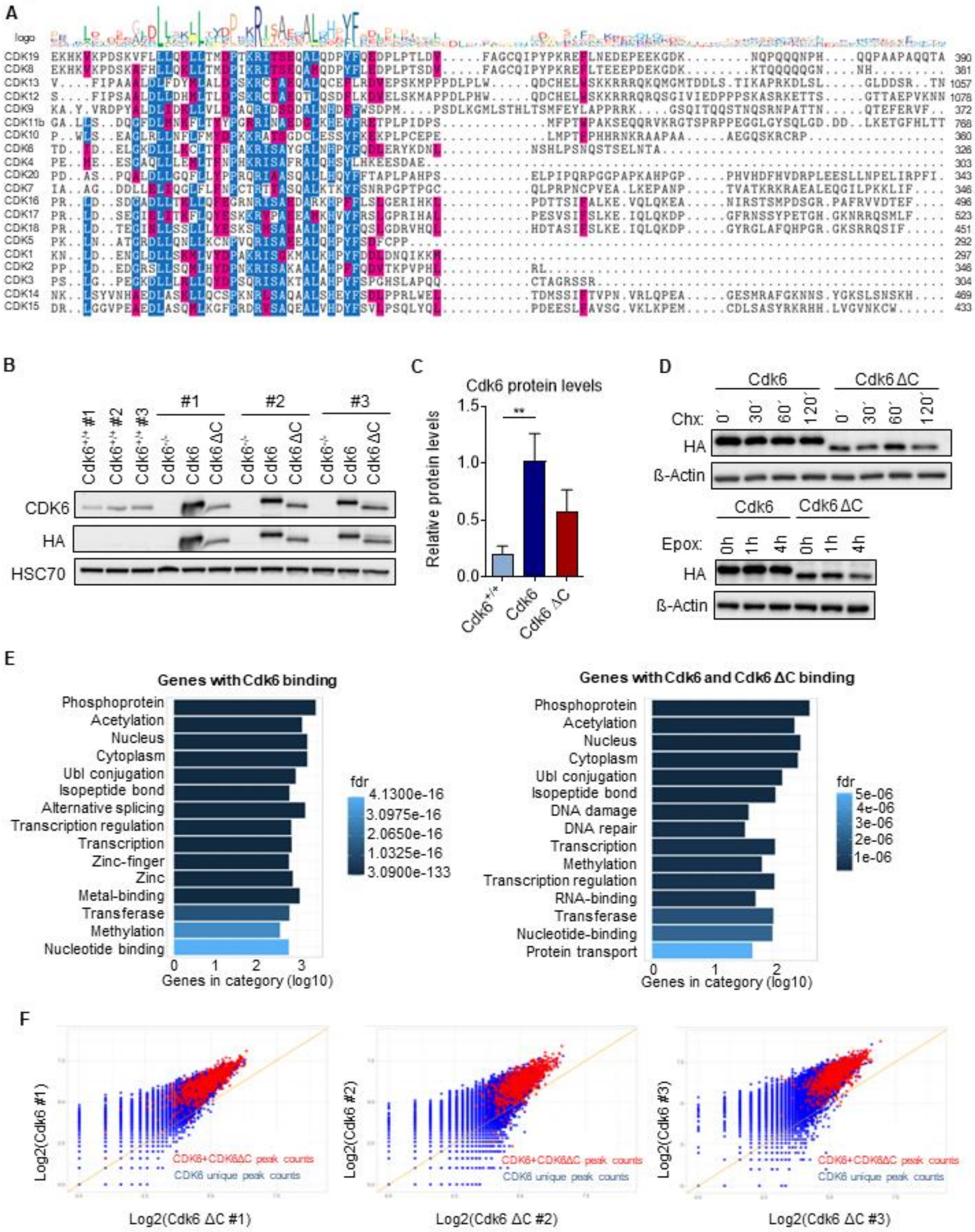
A
B
Cdk6+/+ #1
Cdk6+/+ #2
Cdk6+/+ #3
#1
#2
#3
Cdk6-/-
Cdk6
Cdk6 ΔC
CDK6
HA
HSC70
C
Cdk6 protein levels
Relative protein levels
Cdk6+/+
D
Chx:
0´
30´
60´
120´
ß-Actin
Epox:
0h
1h
4h
E
Genes with Cdk6 binding
Phosphoprotein
Acetylation
Nucleus
Cytoplasm
Ubl conjugation
Isopeptide bond
Alternative splicing
Transcription regulation
Transcription
Zinc-finger
Zinc
Metal-binding
Transferase
Methylation
Nucleotide binding
Genes in category (log10)
fdr
4.1300e-16
3.0975e-16
2.0650e-16
1.0325e-16
3.0900e-133
Genes with Cdk6 and Cdk6 ΔC binding
DNA damage
DNA repair
RNA-binding
Nucleotide-binding
Protein transport
5e-06
4e-06
3e-06
2e-06
1e-06
F
Log2(Cdk6 #1)
Log2(Cdk6 ΔC #1)
Log2(Cdk6 #2)
Log2(Cdk6 ΔC #2)
Log2(Cdk6 #3)
Log2(Cdk6 ΔC #3)
CDK6+CDK6ΔC peak counts
CDK6 unique peak counts

Fig. S1. - The C-terminus of Cdk6 is essential for nuclear localization and chromatin interaction, related to Figure 1

(A) Alignment of the amino acid sequence of the C-terminal domains of the CDKs 1-20. **(B)** Western blot analysis of $Cdk6^{+/+}$, $Cdk6^{-/-}$, Cdk6 and Cdk6 ΔC cell lines for CDK6 and HA (biological replicates #1, #2 and #3). HSC70 was used as loading control. **(C)** Western blot densitometry quantification showing relative Cdk6 protein levels. P values determined by ordinary one-way ANOVA test multiple comparisons (**p=0,004). Error bars show mean ± s.d. **(D)** Cdk6 and Cdk6 ΔC cell lines treated with the translation inhibitor cycloheximide (Chx, 40 µg/ml) or the proteasome inhibitor Epoxomicin (Epox, 10 µM) for the indicated times and followed by western blot analysis for HA. β-actin was used as loading control (representative figure of biological replicate #1). **(E)** Results of an enrichment analysis of uniport keywords in genes with only Cdk6 peaks in their promoters (left) or genes with Cdk6 and Cdk6 ΔC peaks in their promoters (right). **(F)** Scatter plots displaying raw read counts in peaks of a consensus peak set. In blue the peaks that were called in 2 of 3 Cdk6 samples, in red peaks that were called in 2 of 3 Cdk6 ΔC samples. Plots show pairs of Cdk6 and Cdk6 ΔC samples from the same parental cell line.

Fig.S2

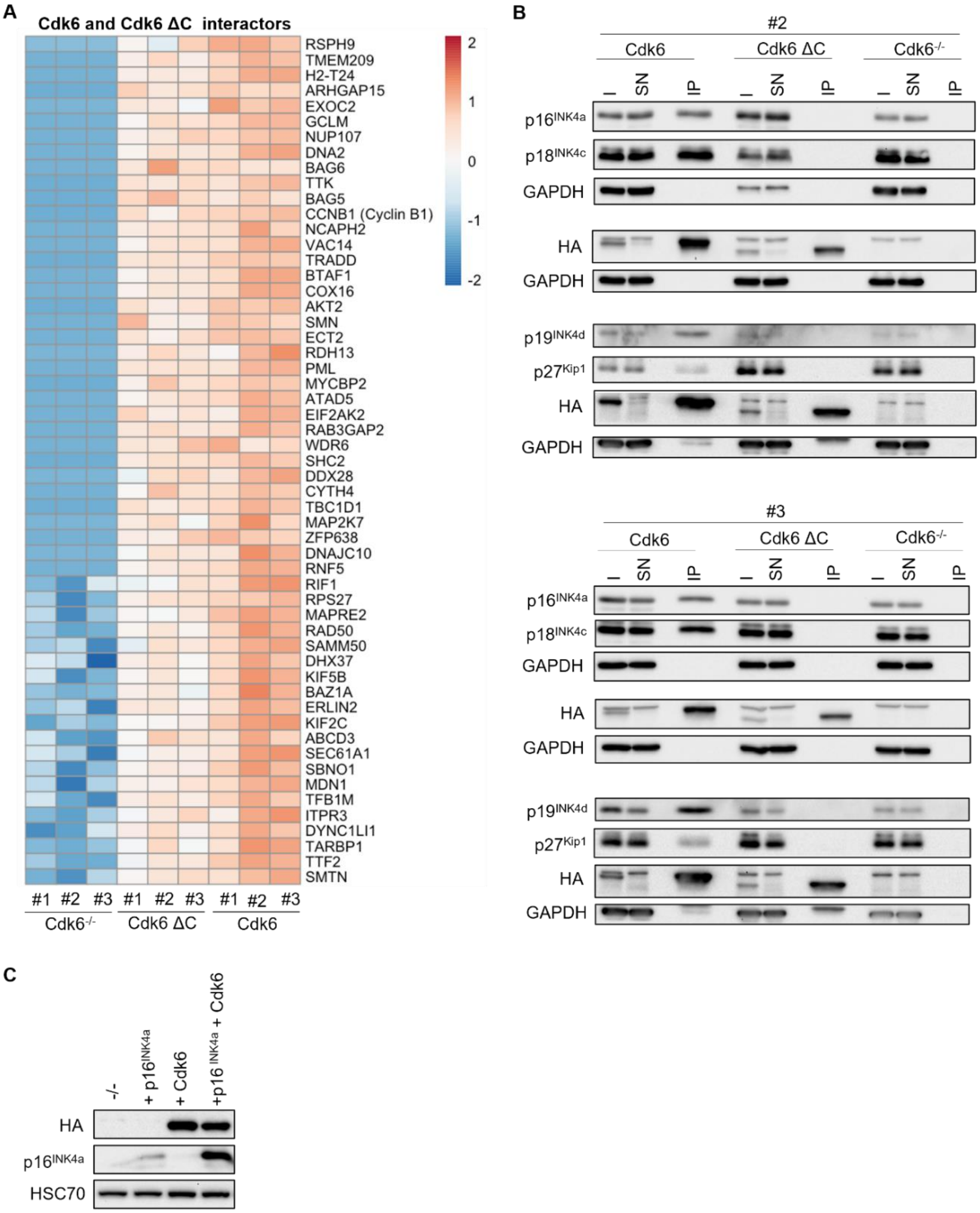
A
Cdk6 and Cdk6 ΔC interactors
RSPH9
TMEM209
H2-T24
ARHGAP15
EXOC2
GCLM
NUP107
DNA2
BAG6
TTK
BAG5
CCNB1 (Cyclin B1)
NCAPH2
VAC14
TRADD
BTAF1
COX16
AKT2
SMN
ECT2
RDH13
PML
MYCBP2
ATAD5
EIF2AK2
RAB3GAP2
WDR6
SHC2
DDX28
CYTH4
TBC1D1
MAP2K7
ZFP638
DNAJC10
RNF5
RIF1
RPS27
MAPRE2
RAD50
SAMM50
DHX37
KIF5B
BAZ1A
ERLIN2
KIF2C
ABCD3
SEC61A1
SBNO1
MDN1
TFB1M
ITPR3
DYNC1LI1
TARBP1
TTF2
SMTN
2
1
0
-1
-2
#1 #2 #3 #1 #2 #3 #1 #2 #3
Cdk6-/-
Cdk6 ΔC
Cdk6
B
#2
Cdk6
Cdk6 ΔC
Cdk6-/-
— SN IP — SN IP — SN IP
p16INK4a
p18INK4c
GAPDH
HA
GAPDH
p19INK4d
p27Kip1
HA
GAPDH
#3
Cdk6
Cdk6 ΔC
Cdk6-/-
— SN IP — SN IP — SN IP
p16INK4a
p18INK4c
GAPDH
HA
GAPDH
p19INK4d
p27Kip1
HA
GAPDH
C
-/-
+ p16INK4a
+ Cdk6
+p16 INK4a + Cdk6
HA
p16INK4a
HSC70

Fig. S2. - CDK6-INK4 interaction is critical for nuclear localization, related to Figure 2

(A) Heatmap showing results from IP-MS experiment to identify Cdk6 and Cdk6 ΔC interaction partners. Scaled log2(abundance) values are plotted for proteins interacting with both, Cdk6 and Cdk6 ΔC. Missing abundance values were replaced by a mock value of ¼ of the smallest measured abundance in the heatmap. **(B)** Anti-HA co-IP from Cdk6 and Cdk6 ΔC cell extracts analysed for $p16^{INK4a}$, $p18^{INK4c}$, $p19^{INK4d}$, $p27^{KIP1}$ and HA. $Cdk6^{-/-}$ cells served as negative control. The input (I), supernatant (SN) and immunoprecipitated (IP) fractions are shown. GAPDH served as loading control. (Biological replicates #2 and #3). **(C)** Western blot analysis of HA and $p16^{INK4a}$ from $p16^{INK4a}/p19^{ARF}/Cdk6^{-/-}$ cell lines reconstituted either with $p16^{INK4a}$, Cdk6 or with both. HSC70 was used as loading control.

Fig. S3

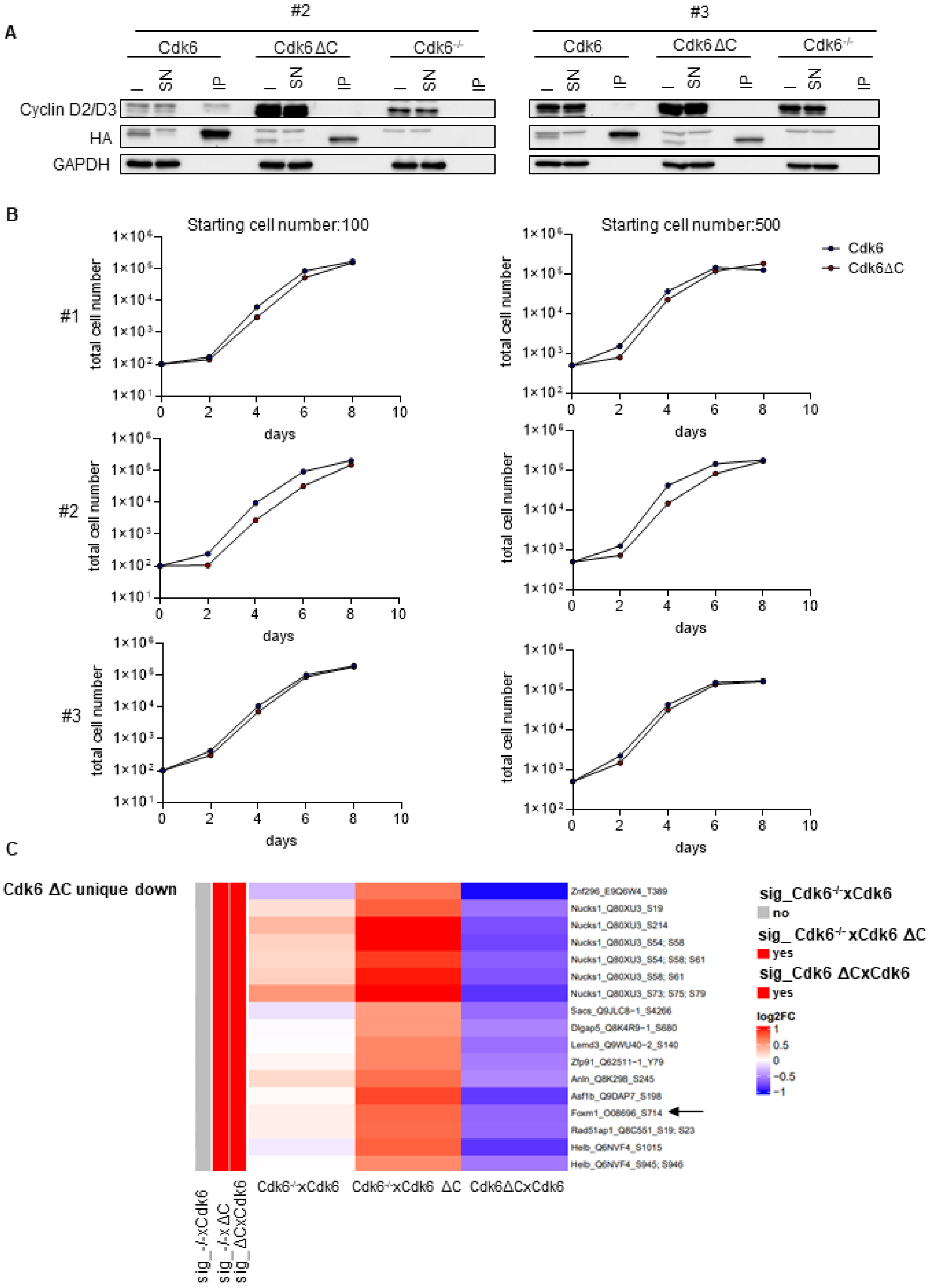
A
#2
#3
Cdk6
Cdk6 ΔC
Cdk6-/-
SN
IP
Cyclin D2/D3
HA
GAPDH
B
Starting cell number:100
Starting cell number:500
Cdk6
Cdk6ΔC
#1
#2
#3
total cell number
days
C
Cdk6 ΔC unique down
Znf296_E9Q6W4_T389
Nucks1_Q80XU3_S19
Nucks1_Q80XU3_S214
Nucks1_Q80XU3_S54; S58
Nucks1_Q80XU3_S54; S58; S61
Nucks1_Q80XU3_S58; S61
Nucks1_Q80XU3_S73; S75; S79
Sacs_Q9JLC8-1_S4266
Dlgap5_Q8K4R9-1_S680
Lemd3_Q9WU40-2_S140
Zfp91_Q62511-1_Y79
Anln_Q8K298_S245
Asf1b_Q9DAP7_S198
Foxm1_O08696_S714
Rad51ap1_Q8C551_S19; S23
Helb_Q6NVF4_S1015
Helb_Q6NVF4_S945; S946
Cdk6-/-xCdk6
Cdk6-/-xCdk6 ΔC
Cdk6ΔCxCdk6
sig_-/-xCdk6
sig_-/-x ΔC
sig_ΔCxCdk6
sig_Cdk6-/-xCdk6
no
sig_ Cdk6-/- xCdk6 ΔC
yes
sig_Cdk6 ΔCxCdk6
yes
log2FC
1
0.5
0
-0.5
-1

Fig. S3. - CDK6 C-terminus is essential for D-type cyclin binding, related to Figure 3

(A) Anti-HA co-IP from Cdk6 and Cdk6 ΔC cells. Cdk6$^{-/-}$ cells served as negative control. The input (I), supernatant (SN) and immunoprecipitated (IP) fractions were immunoblotted for cyclin D2/D3. (n = 2 biological replicates). **(B)** Growth curve analysis of Cdk6 and Cdk6 ΔC cell lines. Either 100 (left) or 500 (right) cells were sorted for each time point and total cell numbers were determined every 48 hours for 8 days (n=3 biological replicates). **(C)** Heatmap showing the list of peptides de-phosphorylated in the context of Cdk6 ΔC.

Fig. S4

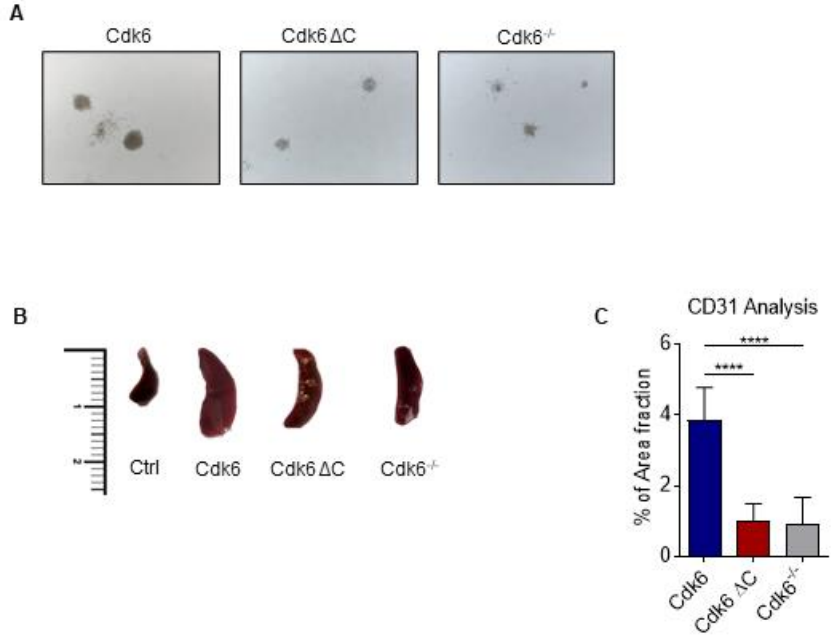

Fig. S4. - The C-terminus of CDK6 is essential for leukemia progression, related to Figure 4

(A) Representative images of colonies 7 days after plating (original magnification x4). **(B)** Representative pictures of spleens of diseased recipient NSG mice transplanted with cell lines expressing either Cdk6, Cdk6 ΔC or no Cdk6. **(C)** Immunofluorescence staining for CD31 was performed to analyze the vascularization of the subcutaneous tumors and quantitative assessment (HistoQuest) of the blood vessels was performed. P values determined by paired one-way ANOVA test (****p<0,0001). Error bars show mean ± s.d.

Fig. S5

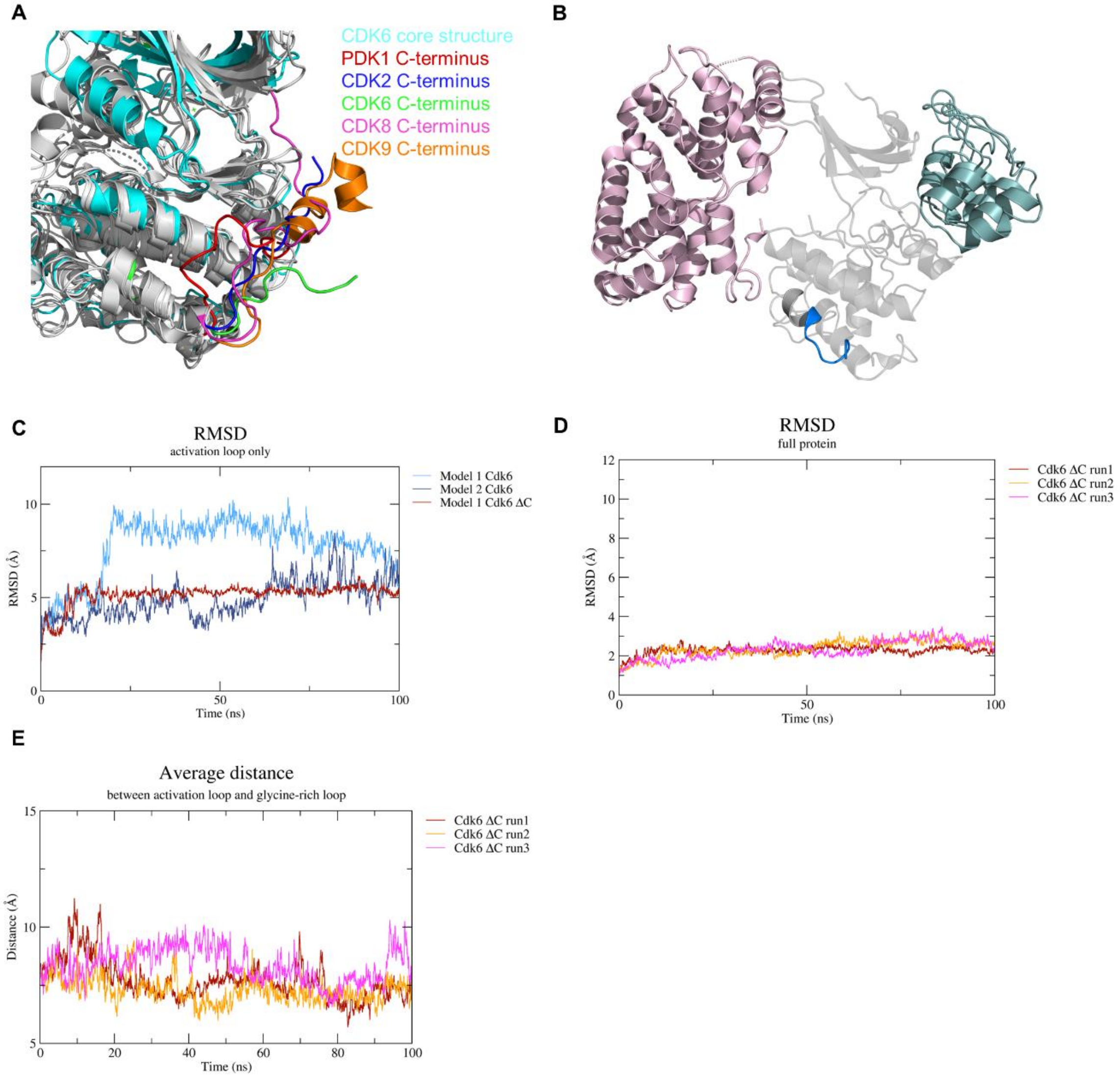

Fig. S5 - The C-terminus of CDK6 determines protein flexibility, related to Figure 5

A) Alignment of different CDK structures revealed loop conformations of their C-termini, with the exception of the helical CDK9 C-terminus (orange, PDB entry 3MIA [S1]). The CDK6 kinase core structure (PDB entry 1BLX [S2]) is depicted in cyan, all other kinase domains are shown in white. The C-termini of the structures are colored according to kinase in red (PDK1, PDB entry 4A06 [S3]), blue (CDK2, PDB entry 3PXF [S4]), green (CDK6, PDB entry 1BLX [S2]), magenta (CDK8, PDB entry 5HBE [S5]) and in orange (CDK9, PDB entry 3MIA [S1]). **(B)** Alignment of Cdk6 (grey) in complex with p16^{INK4a} (light teal, PDB entry 1BI7 [S6]) and V-cyclin (light pink, PDB entry 1XO2 [S7]) shows that the resolved residues of the CDK6 C-terminus (blue, PDB entry 1BI7 [S6]) do not interact with either binding partner. **(C)** The RMSD of the activation loop of Model 1 Cdk6 (light blue) shows the highest deviation of the input structure in

the time course of the MD simulation. Model 2 Cdk6 (dark blue) shows less pronounced deviation but varies more than Model 1 Cdk6 ΔC (red), which shows the most consistent RMSD. **(D-E)** The three MD simulation production runs of Model 1 Cdk6 ΔC retrieved similar results for the RMSD (D) and the average distance between activation loop and glycine-rich loop (E).

Fig.S6

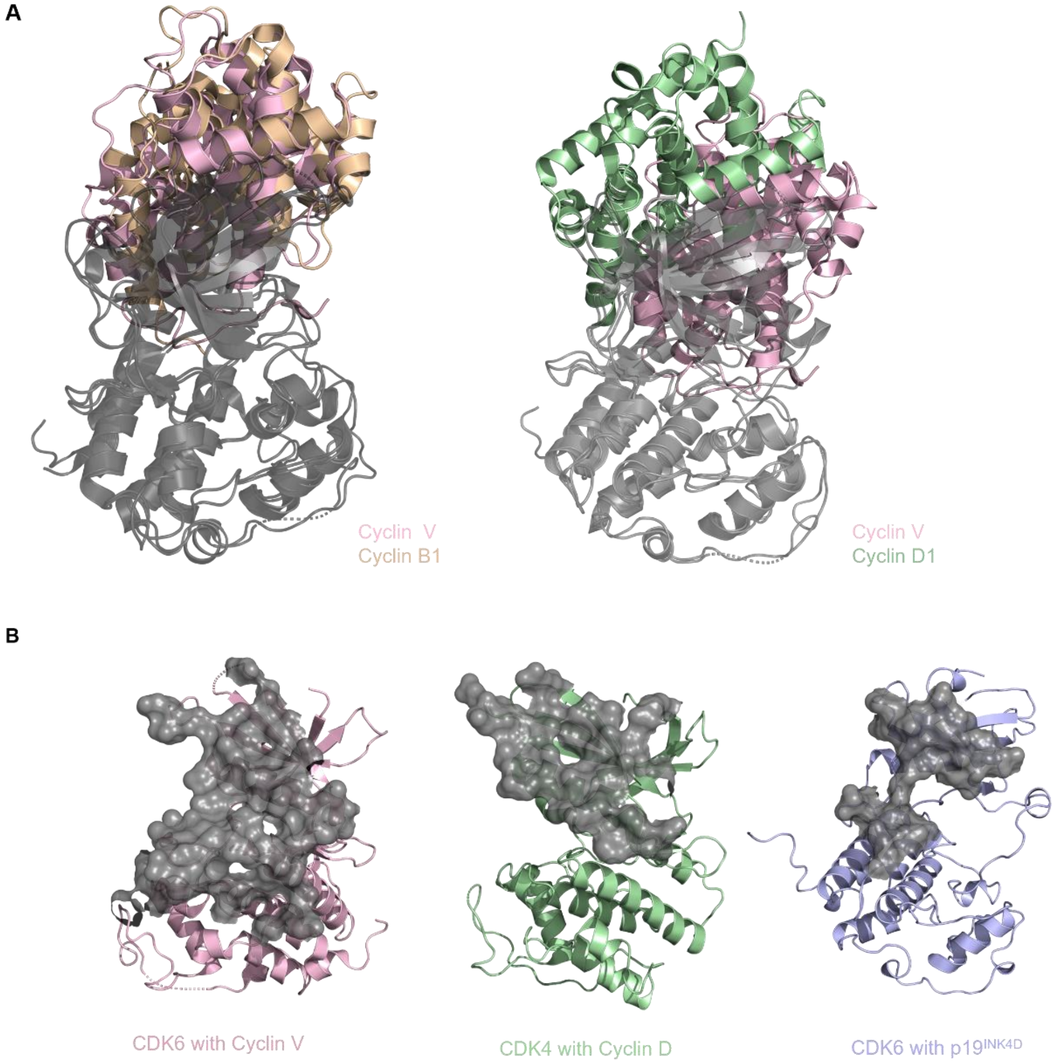

Fig. S6. - Structural analysis between cyclin B1, D1 and V

(A) Alignment of CDK6 in complex with cyclin V (light pink, PDB structure 1XO2 [S7]) against CDK2 bound to cyclin B1 (wheat, PDB entry 2JGZ [S8]) highlights the similar binding modes of the two cyclins. In contrast cyclin D1 (light green, PDB entry 2W9Z [S9]) bound to CDK4 adopts a different binding mode than cyclin V. **(B)** Size comparison of different contact surface areas with different interaction partners. The largest contact surface area is formed between CDK6 (light pink) and cyclin V (PDB entry 1XO2 [S7]) compared to CDK4 (light green) in complex with cyclin D1 (PDB entry 2W9Z [S9]) and CDK6 (light blue) bound to p19 ^{INK4D} (PDB entry 1BLX [S2]).

Fig. S7

A

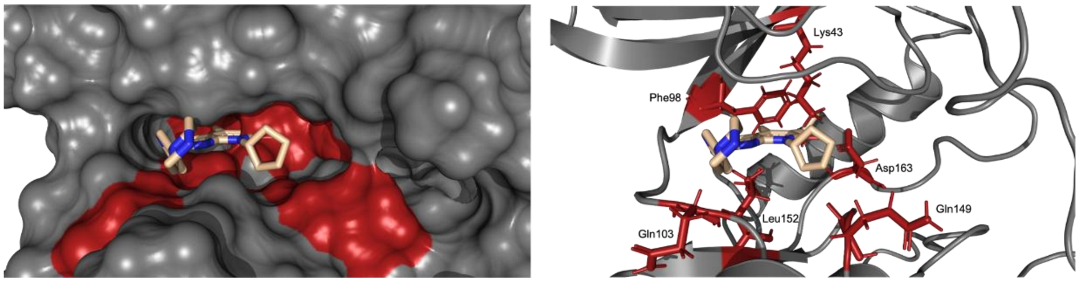

B

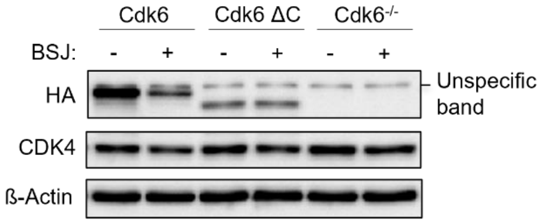

Fig. S7 - Structural analysis of palbociclib binding

(A) Alignment of Model 1 Cdk6 ΔC (grey) with the experimental CDK6-palbociclib complex (wheat sticks, PDB entry 5L2I [S10]) revealed residues that would clash with palbociclib upon binding to Cdk6 ΔC. These are colored in red in the surface depiction on the left and are shown as sticks in the right panel. **(B)** Western blot analysis of Cdk6, Cdk6 ΔC and $Cdk6^{-/-}$ cell lines treated with BSJ-03-123 treatment. β-actin served as loading control.

Supplemental references

1. Tahirov TH, Babayeva ND, Varzavand K, Cooper JJ, Sedore SC, Price DH. Crystal structure of HIV-1 Tat complexed with human P-TEFb. *Nature* (2010) 465:747–751. doi: 10.1038/nature09131

2. Brotherton DH, Dhanaraj V, Wick S, Brizuela L, Domaille PJ, Volyanik E, Xu X, Parisini E, Smith BO, Archer SJ, et al. Crystal structure of the complex of the cyclin D-dependent kinase Cdk6 bound to the cell-cycle inhibitor p19INK4d. *Nature* (1998) 395:244–250. doi: 10.1038/26164

3. Lopez-Garcia LA, Schulze JO, Fröhner W, Zhang H, Süss E, Weber N, Navratil J, Amon S, Hindie V, Zeuzem S, et al. Allosteric regulation of protein kinase PKCζ by the N-terminal C1 domain and small compounds to the PIF-pocket. *Chem Biol* (2011) 18:1463–1473. doi: 10.1016/j.chembiol.2011.08.010

4. Betzi S, Alam R, Martin M, Lubbers DJ, Han H, Jakkaraj SR, Georg GI, Schönbrunn E. Discovery of a potential allosteric ligand binding site in CDK2. *ACS Chem Biol* (2011) 6:492–501. doi: 10.1021/cb100410m

5. Mallinger A, Schiemann K, Rink C, Stieber F, Calderini M, Crumpler S, Stubbs M, Adeniji-Popoola O, Poeschke O, Busch M, et al. Discovery of Potent, Selective, and Orally Bioavailable Small-Molecule Modulators of the Mediator Complex-Associated Kinases CDK8 and CDK19. *J Med Chem* (2016) 59:1078–1101. doi: 10.1021/acs.jmedchem.5b01685

6. Russo AA, Tong L, Lee JO, Jeffrey PD, Pavletich NP. Structural basis for inhibition of the cyclin-dependent kinase Cdk6 by the tumour suppressor p16INK4a. *Nature* (1998) 395:237–243. doi: 10.1038/26155

7. Lu H, Chang DJ, Baratte B, Meijer L, Schulze-Gahmen U. Crystal structure of a human cyclin-dependent kinase 6 complex with a flavonol inhibitor, fisetin. *J Med Chem* (2005) 48:737–743. doi: 10.1021/jm049353p

8. Brown NR, Lowe ED, Petri E, Skamnaki V, Antrobus R, Johnson LN. Cyclin B and cyclin A confer different substrate recognition properties on CDK2. *Cell Cycle* (2007) 6:1350–1359. doi: 10.4161/cc.6.11.4278

9. Day PJ, Cleasby A, Tickle IJ, O'Reilly M, Coyle JE, Holding FP, McMenamin RL, Yon J, Chopra R, Lengauer C, et al. Crystal structure of human CDK4 in complex with a D-type cyclin. *Proc Natl Acad Sci U S A* (2009) 106:4166–4170. doi: 10.1073/pnas.0809645106

10. Chen P, Lee NV, Hu W, Xu M, Ferre RA, Lam H, Bergqvist S, Solowiej J, Diehl W, He Y-A, et al. Spectrum and Degree of CDK Drug Interactions Predicts Clinical

Performance. *Mol Cancer Ther* (2016) 15:2273–2281. doi: 10.1158/1535-7163.MCT-16-0300
